# Turmeric-derived nanovesicles as novel nanobiologics for targeted therapy of ulcerative colitis

**DOI:** 10.7150/thno.73650

**Published:** 2022-07-18

**Authors:** Caifang Gao, Yangyang Zhou, Zhejie Chen, Hongyi Li, Yaqin Xiao, Wei Hao, Ying Zhu, Chi Teng Vong, Mohamed A. Farag, Yitao Wang, Shengpeng Wang

**Affiliations:** 1 State Key Laboratory of Quality Research in Chinese Medicine, Institute of Chinese Medical Sciences, University of Macau, Macao, China; 2 Department of Integrative Oncology, Fudan University, Shanghai Cancer Center, Shanghai 200032, China; 3 Pharmacognosy Department, College of Pharmacy, Cairo University, Kasr el Aini St., Cairo 11562, Egypt

**Keywords:** Turmeric-derived nanovesicles, Ulcerative colitis, Gut microbiota, Macrophage polarization, Fresh herbs

## Abstract

**Rationale**: Ulcerative colitis (UC), a typical kind of inflammatory bowel disease (IBD), is an idiopathic chronic intestinal inflammation. Conventional therapeutic strategies mainly focus on the rebalance of pro-inflammation and anti-inflammation cytokines, whereas targeting damaged intestinal barriers, imbalanced intestinal microbiota and dysregulated mucosal immune responses in UC remain a big challenge. The objective of this study was to develop turmeric-derived nanovesicles (TNVs) for alleviation of colitis and explore the underlying mechanisms.

**Methods**: TNVs were isolated and purified through differential centrifugation. The targeted ability was evaluated on the dextran sulfate sodium (DSS)-induced mouse model by IVIS imaging system. The anti-inflammation efficacy was studied in lipopolysaccharide (LPS)-induced macrophages and DSS-induced acute and chronic colitic mouse model. In addition, the influence of TNVs on the intestinal microbiota was investigated via 16S rRNA microbiome sequence and the condition of macrophage polarization after TNVs treatment was analyzed by flow cytometry.

**Results**: TNVs were isolated and characterized as nano-size spheroids. The IVIS imaging experiment indicated that orally administrated TNVs could accumulate in the inflamed colon sites and exhibited superior anti-inflammatory activity both *in vitro* and *in vivo*. The 16S rRNA sequencing suggested the important role of TNVs in the regulation of gut microbiota. Further, TNVs could promote the transformation of M1 phenotype to M2 macrophages and restore the damaged intestinal epithelium barrier to exert the anti-colitis efficacy.

**Conclusion**: Collectively, oral administration of TNVs exhibited excellent anti-inflammatory efficacy through restoring the damaged intestinal barrier, regulating the gut microbiota and reshaping the macrophage phenotype. This study sheds light on the application of natural exosome-like nanovesicles for the treatment of UC.

## Introduction

Ulcerative colitis (UC), a main type of inflammatory bowel disease (IBD), represents a chronic and debilitating intestinal inflammatory condition 1. The incidence of UC has continued to increase worldwide and has become a global health problem 2. Generally, genetic, environmental, microbial and immunological factors are attributed to the pathogenesis of UC 3. Patients with UC usually manifest abdominal pain, weight loss, diarrhea, and even hematochezia. Current therapeutic options compose of amino-salicylic acids (ASA), glucocorticoids, immunosuppressants and emerging biological therapies, typically including anti-TNF (tumor necrosis factor) agents, proinflammatory cytokines inhibitors, anti-adhesion agents, and downstream signaling inhibitors 4. Although these medications could relieve the symptoms of UC patients, drug resistance and their side effects, such as gastrointestinal adverse reactions, autoimmune disorders, dermatological diseases, and even malignancies, seriously limit their medical application 5-7. Therefore, the discovery and development of safe, effective and low-cost drugs are urgently needed for UC patients.

Novel drug delivery systems have been increasingly explored for the targeted therapy of UC focusing on the different factor changes during the pathological condition 8, 9. However, their biosafety evaluation and industrial large-scale production still require extensive investigations to be applied in clinical 10. Recently, exosomes, a type of nano-size membrane vesicles secreted into extracellular space, have attracted extensive investigations 11, 12. Cumulative evidence has suggested that exosomes perform various biological activities particularly in cells communication and that several bioactive agents in exosomes, such as proteins and microRNAs, are identified to be closely related with the pathogenesis of most human diseases, of potential to be used in disease diagnosis, prognosis, and treatment 13-15. Nevertheless, the production and application of animal/cell origin-derived exosomes are still facing obstacles due to the limited resources and immunogenicity 16. Recently, several plant-derived exosomes-like nanoparticles have been discovered and reported to mediate for interspecies communication and exert potential anti-cancer and anti-inflammation properties 17-23. Previous studies indicate that the utilization of plants as nano-factories for the preparation of medical exosomes could provide a novel and facile platform for management of diseases.

Fresh herbs, as a characteristic and special form of Chinese medicine, refer to raw materials without any processing. Many fresh herbs possess stronger function or unique properties than that of dried herbs 24, 25. Fresh herbs have abundant resource and long-term clinical application experience. Recently, exosome-like nanoparticles derived from ginger and ginseng have been reported to alleviate inflammatory diseases, inhibit the proliferation of tumor and promote the differentiation of stem cells 18, 21, 26. Thus, the combination of fresh herbs and emerging exosomes may bring a safe and promising approach for the treatment of several ailments including UC.

Turmeric (*Curcuma longa* L.), a famous medicinal and food dual-purpose Chinese medicine, has received increasing attention for management of UC 27, 28. Since the enrichment of bioactive molecules in the plant-derived exosome-like nanoparticles, we speculate that turmeric-derived nanovesicles (TNVs) may be utilized as a novel therapeutic for several diseases. Herein, exosome-like nanovesicles were isolated from fresh turmeric, and their anti-inflammatory activities and underlying mechanisms were investigated. This study sheds light on the application of natural exosome-like nanovesicles in the treatment of UC *via* oral route, which opens the door for the treatment of other disorders.

## Results

### Characterization of TNVs from fresh turmeric rhizome

**Figure 1:**
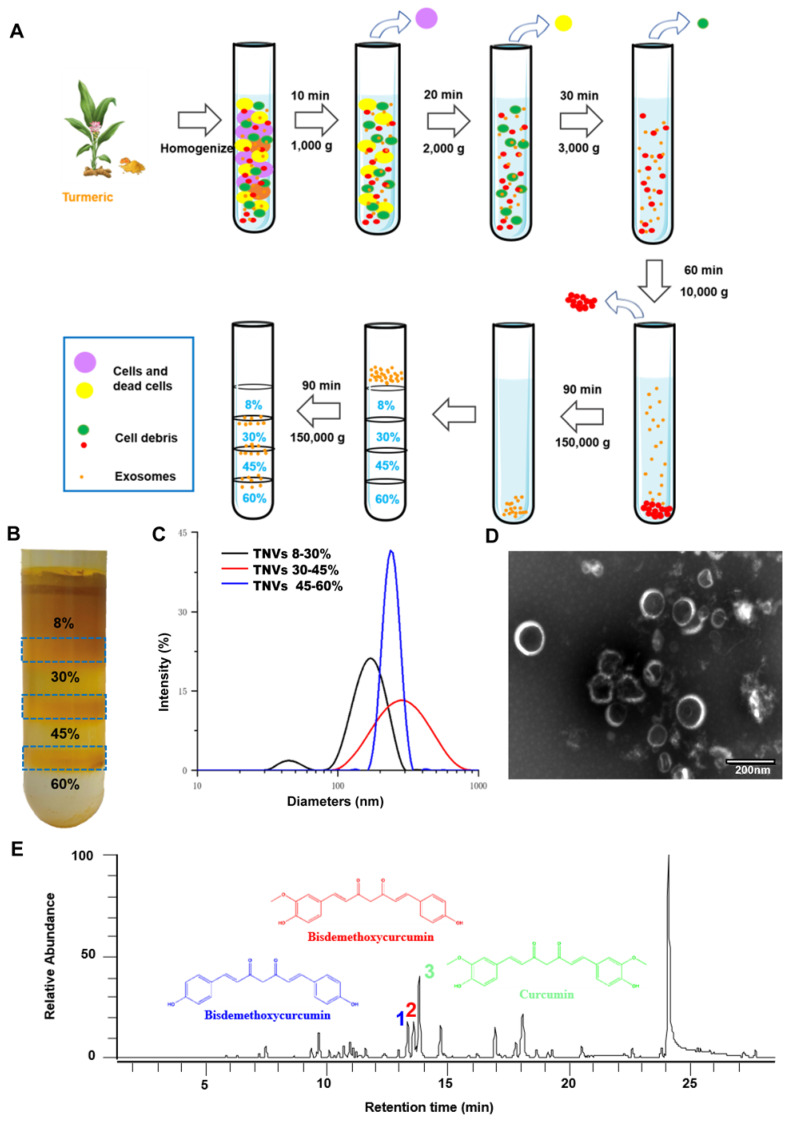
**Isolation and characterization of turmeric-derived nanovesicles (TNVs). A.** The isolation and purification scheme. **B.** The representative image of purified TNVs after sucrose gradient centrifugation. **C.** The size distribution of TNVs from various bands between different sucrose concentration. **D.** The transmission electron microscopy of TNVs between 30-45% sucrose solution. **E.** The LC-MS spectrum of curcuminoids in TNVs.

TNVs were isolated from fresh turmeric juice and purified using sucrose gradient ultracentrifugation (Figure 1A). As shown in Figure 1B, TNVs mainly distributed at the 8/30% and 30/45% interfaces of the sucrose gradient, whereas a faint band was observed at the interface of 45/60%. The size distributions, polydispersity index (PDI) and zeta potentials of purified TNVs were measured using dynamic light scattering (DLS). The diameters were 191.7 ± 15.8, 243.9 ± 13.9 and 800.5 ± 66.2 nm from up to the bottom sucrose interface, respectively (Figure 1C). The zeta potentials were about -15.0 mV in PBS solution (Table S1). To observe the morphology of TNVs, TNVs at proper concentration were negatively stained with 1% uranyl acetate solution and captured by transmission electron microscope (TEM). The result showed a uniform spherical structure and the size was consistent with those measured by DLS (Figure 1D). Based on these findings, TNVs located at the 45/60% interface were excluded from further analyses due to their lower yield and large size. Importantly, the yield of TNVs located at the 8/30% and 30/45% interfaces can be up to 100 mg and 50 mg per 1000 g fresh turmeric (Table S2). A consistent yield was observed among different turmeric batches, of course which all originated from Qianwei, Sichuan, China, and it represented a high-level of TNVs production compared to the synthesis of nanoparticles. Although regardless of the yield and size distribution, TNVs of 8/30% and 30/45% interfaces almost had no significant difference, the targeted ability was further evaluated in a colitic mouse model.

Lipids compositional analysis using liquid chromatography-mass spectrometry (LC-MS) showed that TNVs was mainly comprised of fatty acids (FA), diacylglycerols (DAG), triacylglycerols (TAG) phosphatidylcholine (PC), and phosphatidyl ethanolamine (PE) (Figure S1A), which are common lipid components and not specific to turmeric. Based on the existing liposome delivery system, various lipids are important to assist the formation of spheroids and the entrapment of drugs in different properties liposomes. We speculated that these lipids in TNVs greatly contributed to the formation of nanovesicles and to maintain its sphere-like structure.

Next, protein composition was determined using LC-MS/MS. The molecular weight of these proteins ranged from 4 to 26 kDa. Gene Ontology (GO) database (Figure S1B) revealed that the proteins contained in TNVs corresponded to proteins involved in the molecular functions, cellular components, and biological processes. In addition, about 70% proteins of TNVs played a pivotal role in the metabolic pathways and the biosynthesis of secondary metabolites according to Kyoto Encyclopedia of Genes and Genomes (KEGG) annotations (Figure S1C).

It is well known that dried turmeric contains large amounts of curcuminoids, especially curcumin, demethoxycurcumin and bisdemethoxycurcumin, which exerted superior anti-oxidant, anti-inflammatory and anti-cancer efficacy. Therefore, the contents of these three compounds in the TNVs were measured by HPLC-MS (mass spectrum was shown in Figure 1E) and the results are presented in Table S3. Compared to the dried turmeric, the contents were too low to exert a biological function. Due to the poor solubility of curcuminoids and the difference between fresh and dried medicinal materials, the active components of TNVs should be explored further.

### *In vivo* distribution of TNVs

**Figure 2:**
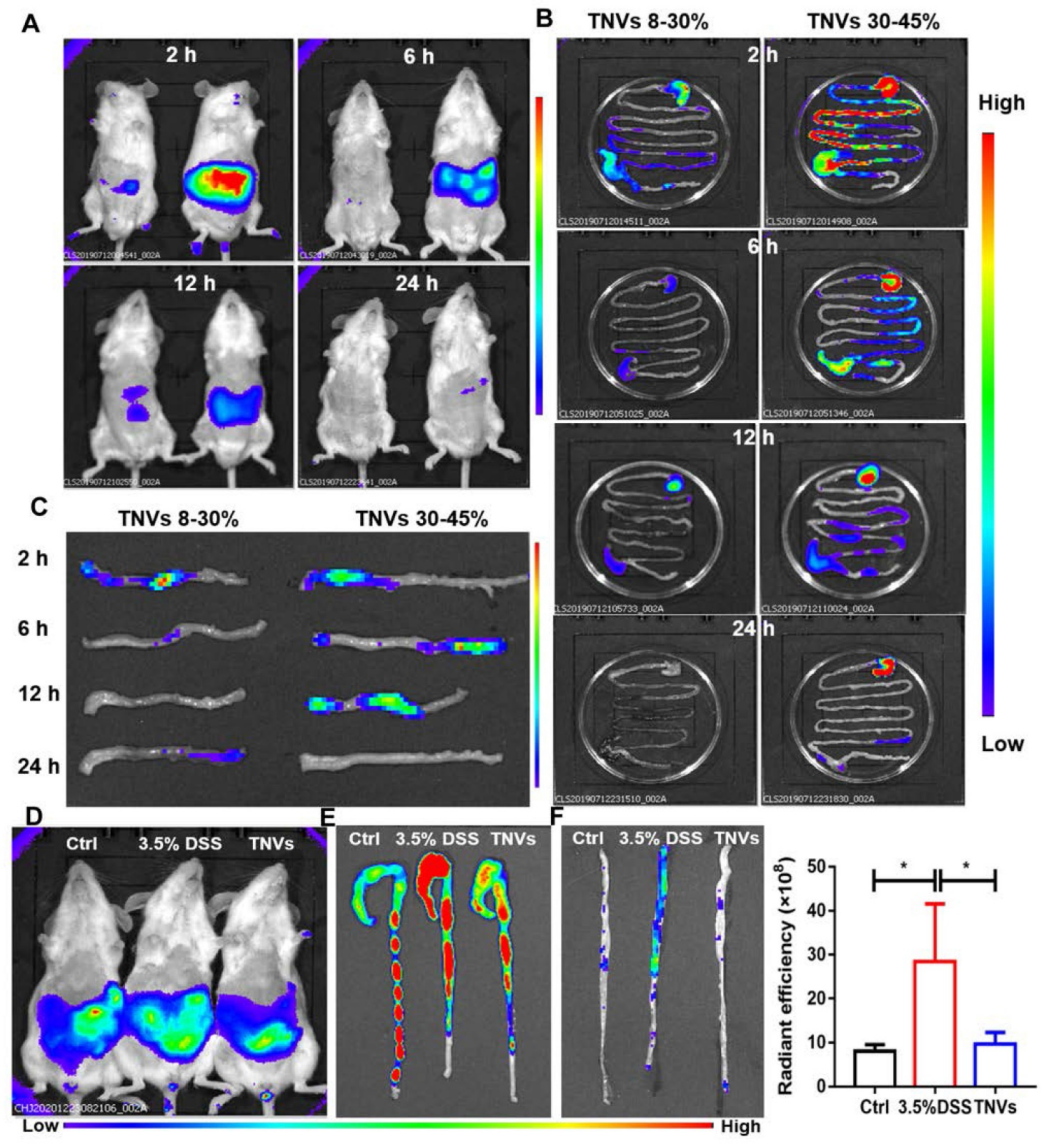
***In vivo* and* ex vivo* distribution of DiR-labeled TNVs. A.** Whole body imaging of the mice at 2, 6, 12 and 24 h after oral administration of TNVs originated from 8%-30% (left) and 30%-45% (right) band. **B.** The distribution of DiR-labeled TNVs in the gastrointestinal tract at different time points. **C.** The distribution of DiR-labeled TNVs in the distal colons at different time point. **D.** Whole body imaging of the mice (healthy, DSS-induced and TNVs treated) at 6 h with DiR-labeled TNVs treatment. **E-F.** The accumulation and radiant efficiency statistics of DiR-labeled TNVs in the feces-contained and no-feces colon dissected from mice at different condition. Data are shown as mean ± SD, n = 3. Significance as *P < 0.05.

To investigate the biodistribution of TNVs, 3.5% DSS were given to induce acute colitis mice for 7 days. Upon successful establishment of the mouse model, fasted mice were orally given a single dose of DiR-labeled TNVs (0.3 mg/mL). The fluorescence intensity in mice was imaged, the mice were killed, and colon tissues were harvested for fluorescence imaging using an IVIS Spectrum Series imaging system at different time points after gavage (2, 6, 12, and 24 h). As shown in Figure 2A, the fluorescence intensity of TNVs reached the maximum after intragastric administration for 6 h, and then gradually declined, and even disappeared (Figure 2B-C). The distribution of TNVs 30-45% in the colon was more obvious than TNVs 8-30% at the any timepoint, so the subsequent experiments were only carried out for TNVs 30-45% and the name of TNVs in the context is referred to TNVs 30-45%. The stability of TNVs in the in the stimulated gastric and intestinal fluids were further conducted. As shown in Figure S2, the size and PDI of TNVs in PBS, stimulated gastric and intestinal fluids were 248.2 nm (0.165), 254.7 nm (0.208) and 341.1 nm (0.280), respectively. The data indicated that the size and PDI of TNVs barely changed and that TNVs could keep stable in the harsh gastric and intestinal environment.

Next, another distribution experiment was conducted to observe the targeting ability and anti-colitis activities of TNVs. Mice were randomly divided into three groups: normal group, model group (3.5% DSS for 7 days) and TNVs therapeutic group (TNVs + 3.5% DSS for 7 days). At day 8, DiR-labeled TNVs was orally administered to the mice. After 6 h, mice were imaged using an IVIS Spectrum Series system and the fluorescence intensity of model mice was the highest. The fluorescence intensities of the *ex-vivo* colons were consistent to that of the abdominal section, regardless of colons with feces or not (Figure 2D-F). Taken together, TNVs appear to selectively accumulate in the inflamed colon lesion to exert their targeting-inflammation and anti-colitis activity.

### *In vitro* cellular uptake and endocytosis mechanisms of TNVs

Cellular uptake is a key process of drug delivery systems to exhibit their function. The data in Figure S3A-C revealed that the fluorescence intensity became higher in Raw 264.7 macrophages and NCM 460 cells treated with DiO-labeled TNVs as the co-incubation time increased. In addition, the fluorescence intensity of LPS-induced group was stronger than that of control group (Figure S4), indicating that TNVs could target the inflammatory cells.

**Figure 3:**
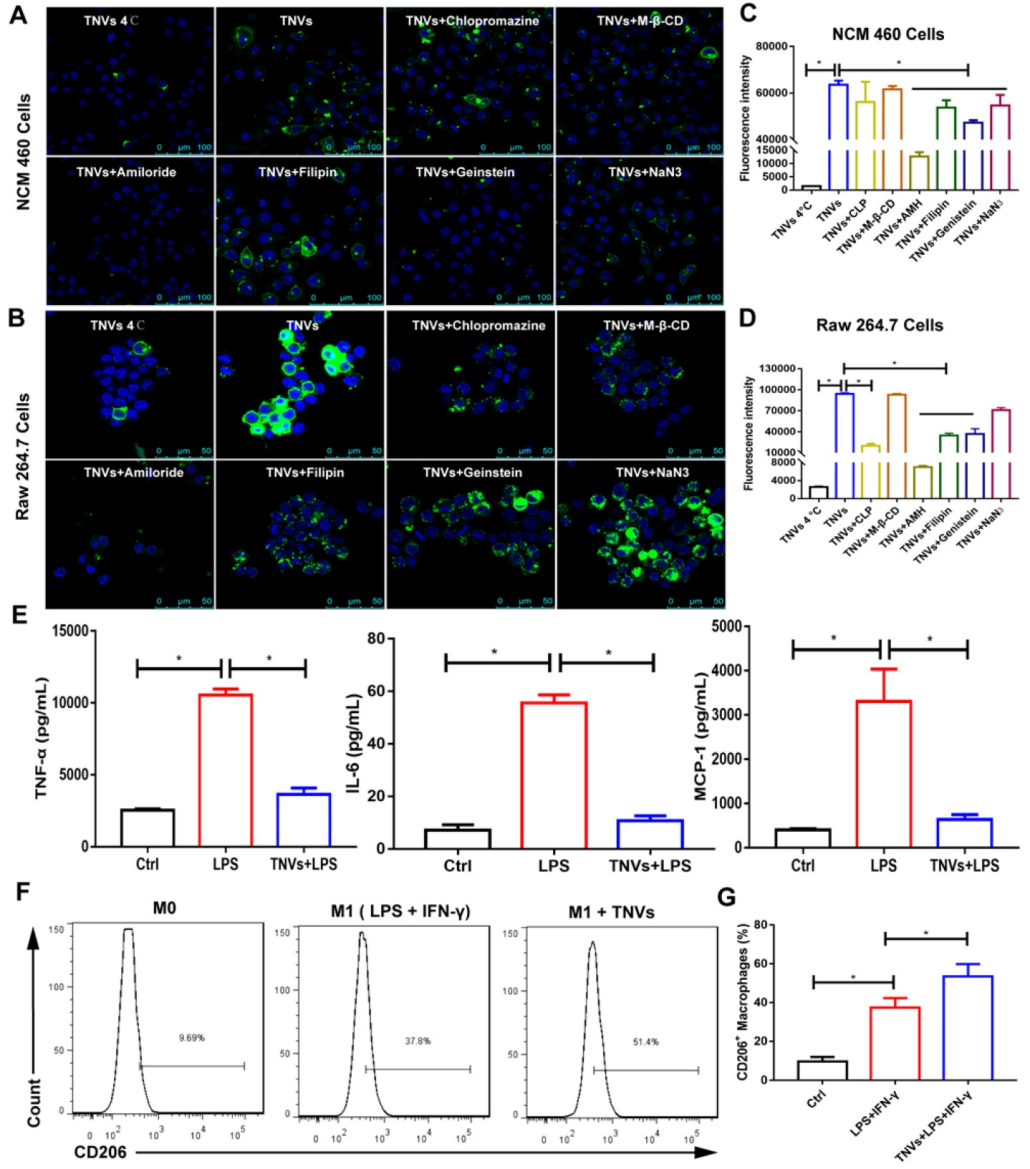
***In vitro* cell internalization mechanisms, anti-inflammatory properties and the effect on the macrophage polarization of TNVs. A-B.** Representative fluorescence images of cellular uptake of DiO-labeled TNVs in NCM 460 cells and Raw 264.7 macrophages after co-incubation for 6 h and additional different inhibitors treatment 1 h. **C-D.** Fluorescence intensity of cellular uptake of DiO-labeled TNVs in NCM 460 cells and Raw 264.7 macrophages determined using flow cytometry. **E.** *In vitro* anti-inflammatory activities of TNVs on Raw 264.7 macrophages. The concentrations of pro-inflammatory cytokines (TNF-α, IL-6, and MCP-1) were quantified using ELISA assay. **F-G.** Flowcytometry analysis of CD206 expression in Raw 264.7 macrophages. Data are shown as mean ± SD, n = 3. Significance as *P < 0.05.

To confirm cellular uptake mechanism of TNVs in Raw 264.7 macrophages and NCM 460 cells, both cells were treated with various endocytosis inhibitors, including caveolae-mediated endocytosis inhibitors (Genistein and Filipin), clathrin-mediated endocytosis inhibitor (chlorpromazine, CLP), macro-pinocytosis inhibitor (amiloride hydrochloride, AMH), cholesterol clearance agent (methyl-beta-cyclodextrin, M-β-CD), and an energy depletion agent (NaN_3_). Meanwhile, the effect of low temperature on the cellular uptake was also studied and both cells were incubated with DiO-labeled TNVs at 4°C for 6 h. As shown in Figure 3A-D, the cellular uptake of TNVs in Raw 264.7 macrophages and NCM 460 intestinal epithelium cells was affected by most of the inhibitors and was significantly suppressed by AMH, Genistein and Filipin. Low temperature treatment also greatly decreased the cellular uptake in both cells. The effect of CLP in Raw 264.7 cells was larger than that in NCM 460 cells likely attributed to the heterogeneity of cell surface composition. NaN3, a common ATP depletion agent, exhibited more obvious efficacy in NCM 460 cells than Raw 264.7 cells, which might be associated with the phagocytosis feature of macrophages. However, the M-*β*-CD hardly affected the cellular uptake of both cells. These results indicated that TNVs uptake by Raw 264.7 macrophages and NCM 460 intestinal epithelium cells were multifaceted.

### *In vitro* anti-inflammatory activity and macrophage polarization effects of TNVs

Many typical pro-inflammatory cytokines involved in the pathogenesis of UC both in mice and patients 29. These cytokines are mainly produced and secreted by macrophages. To assess the anti-inflammatory activity, Raw 264.7 cells were stimulated with LPS or co-treated with TNVs and LPS. The levels of TNF-α, Interleukin 6 (IL-6), and Monocyte Chemoattractant Protein 1 (MCP-1) elevated by LPS were greatly reduced by TNVs, almost to the level of normal group (Figure 3E), suggesting that TNVs could provide a green and efficacious approach for the management of inflammatory disorders.

As macrophage polarization is a critical factor during inflammatory conditions and many cancers 30, the effect of TNVs on the macrophage polarization was assessed in Raw 264.7 cells. FACS results (Figure 3F-G) showed that the cluster of differentiation (CD)206^+^ (the typical marker of M2 macrophages) rate increased from 37.8% to 51.4% after TNVs treatment in LPS- and IFN-γ-stimulated F4/80^+^CD11b^+^ macrophages. The statistics in Figure 3G also indicated higher CD206 positive rate in the treatment group. Collectively, the inflammatory status could be adjusted by TNVs through promoting macrophage to differentiate into M2 phenotype.

### TNVs ameliorated colitis-related symptoms of DSS-induced acute colitis

**Figure 4:**
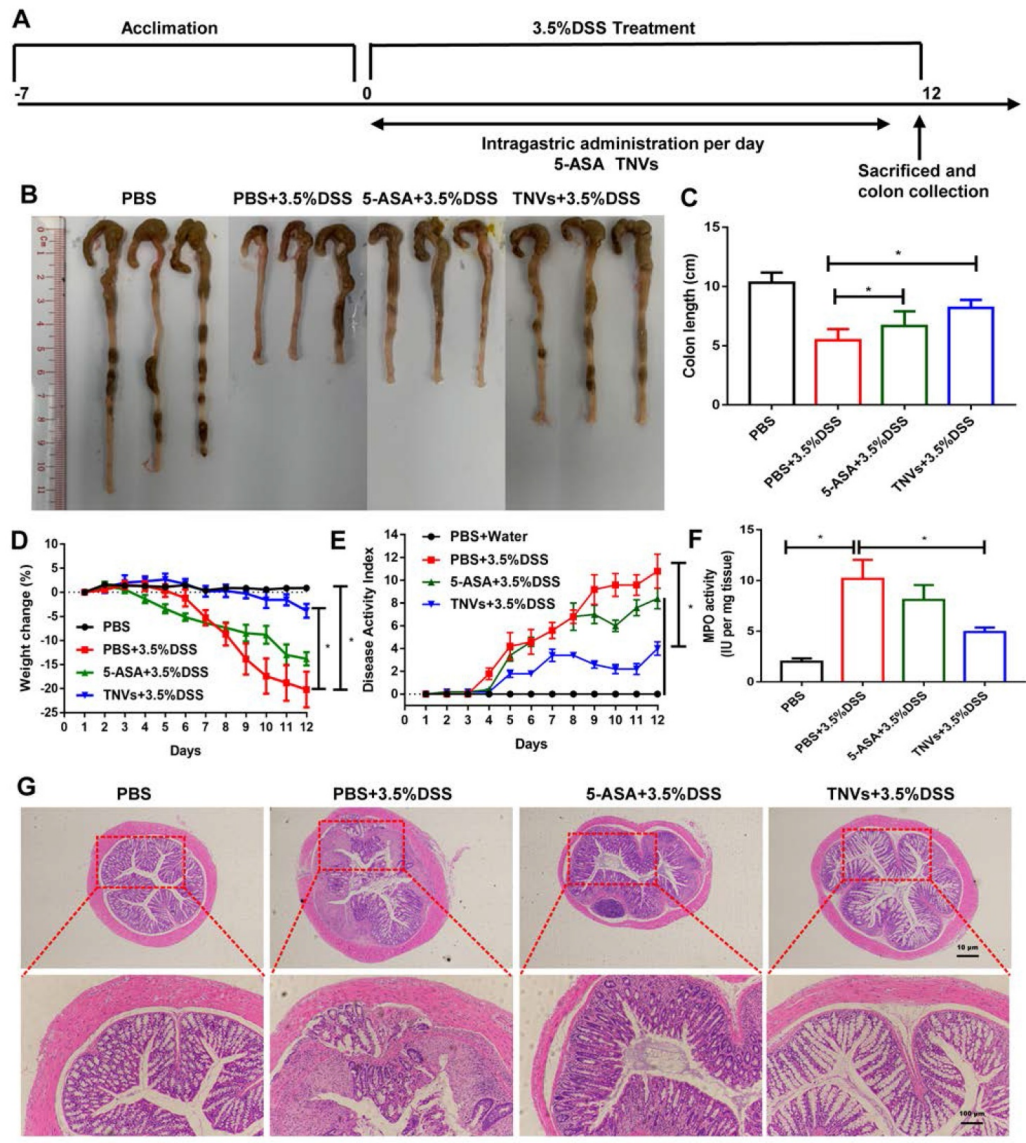
** TNVs alleviated colitis-related symptoms in DSS-induced acute colitic mice. A**. The flow chart represents the establishment of mice colitis model and drug treatment design. **B-C.** Representative images of colons and average colon length from different groups. **D.** Body weight change expressed as the percentage of the day-zero weight. **E.** DAI, **F.** Colonic MPO activity, **G.** Representative histological sections of distal colons stained with H&E (4× and 10× magnification). Data shown as means ± SD, n = 8. The principle of determining disease activity index (DAI) is the summation of the body weight loss (0- <1%, 1- 1~5%, 2- 5~10%, 3- 10~20%, 4- > 20%), stool consistency (0- normal, 2- loose, 4- diarrhea) and rectal bleeding (0- normal, 2- positive finding, 4- gross bleeding).

Guided by the *in vitro* results, the therapeutic efficacy of TNVs against UC was further investigated. The scheme for the experimental design of TNVs against acute colitis was shown in Figure 4A. Due to the mucosal damage, intestinal epithelium barrier disruption and dehydration in the development of UC, colon shortening is a critical parameter for the evaluation of the severity of inflammation 31. From Figure 4B-C, difference in colon length in the healthy group versus DSS-challenged group was observed at 9.3 ± 0.85 and 5.5 ± 0.94 cm, respectively. The positive control (5-ASA) group had a longer colon length (6.7 ± 0.43 cm), indicating the therapeutic value of conventional non-steroidal anti-inflammatory drugs against colitis. The colon length of TNVs treatment group (8.2 ± 0.56 cm) was much longer than that of the 5-ASA group, suggesting the excellent protection ability of TNVs from intestinal mucosa integrity. In Figure 4D, we can see that the body weight of mice in the healthy group was steady, whereas mice of the DSS-induced group suffered from serious weight loss (nearly 20% of the initial weight). Surprisingly, weight loss was mitigated after 5-ASA and TNVs treatment, especially the TNVs treatment group (less than 5%). The disease activity index (DAI), the scoring system that according to the degree of weight loss, stool consistency, and fecal bleeding, reflects the activity of colitis in clinical 32. The highest DAI score in the DSS-induced mice group was presented in Figure 4E and the DAI of the TNVs-treated group was much lower than that of the DSS and 5-ASA groups. Myeloperoxidase (MPO), a lysosomal protein in neutrophils, is a key indicator in promoting neutrophil infiltration into colon tissues during the intestinal inflammation 33. TNVs could significantly reduce the MPO activity elevated by DSS exposure (Figure 4F). Finally, histological change was performed via hematoxylin-eosin (H&E) staining of colon tissues. The colon tissues of healthy mice have casual inflammatory cells and intact epithelium structure. After treatment with DSS, abundant infiltration of inflammatory cells, destruction of crypt structures, and great loss of goblet cells were observed (Figure 4G). The colon tissues from the TNVs-treated group, showed an obvious decrease in inflammation-related features.

### TNVs inhibited macrophage infiltration and altered macrophage polarization

**Figure 5:**
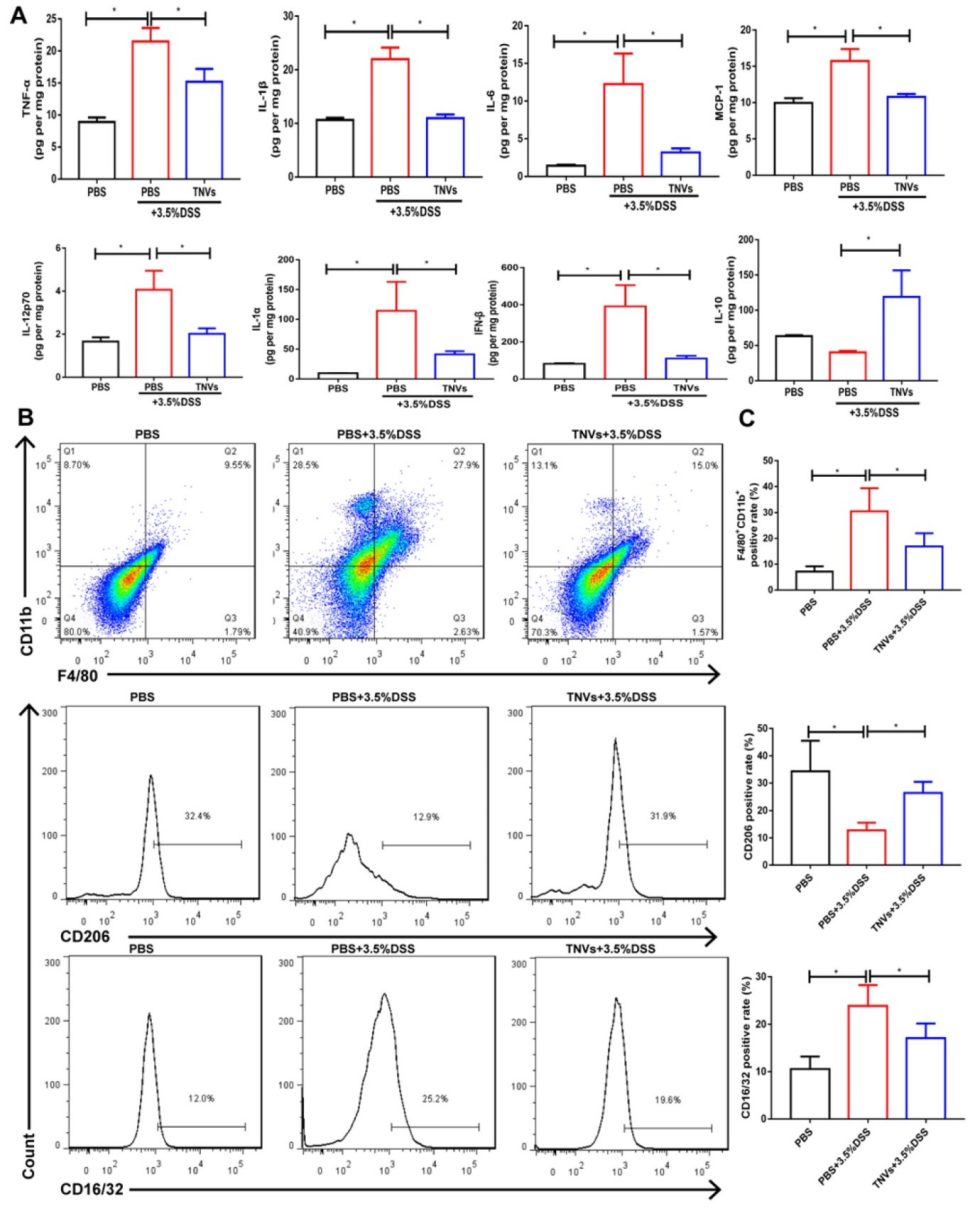
** TNVs mitigated intestinal inflammation responses and reshaped macrophage differentiation in colonic lamina propria. A.** Cytokine productions of TNF-α, IL-1β, IL-6, MCP-1, IL-12p70, IL-1α, IFN-β and IL-10 in colon tissues. **B-C.** The FACS analysis of F4/80^+^CD11b^+^macrophage and the positive rate of CD16/32 and CD206 gated on F4/80^+^CD11b^+^ macrophages from colonic lamina propria in different groups. Data are shown as means ± SD; n = 5. Significance as *P < 0.05.

During UC, neutrophils are typically activated in the inflamed intestinal site 34. This process can be followed by increased levels of typical pro-inflammatory cytokines (IL-1β, MCP-1, TNF-α, IL-6, IL-12p70, IL-1α and IFN-β) in the DSS group compared to healthy mice (Figure 5A). Orally administered TNVs could noticeably decrease the secretion of these above cytokines. IL-10 is a well-recognized anti-inflammatory cytokine, the levels of which were much higher in the TNVs-treated group compared in the DSS group.

Immune cells play a critical role and promote severe lesions during UC 35. As TNVs were found to promote M1 macrophage transformation into M2 phenotype in Raw 264.7 cells, the differentiation of macrophages in the colon tissue were studied in acute colitis mice. As seen in Figure 5B-C, the population of F4/80^+^ CD11b^+^macrophage from colonic lamina propria was increased in DSS group mice (27.9%). As expected, TNVs treatment group (15.0%) prominently reduced the population of activated macrophages. More importantly, TNVs treatment decreased the positive cell rate of CD16/32, a typical intestinal M1 macrophage marker, and increased positive cell rate of CD206, gated on F4/80^+^ CD11b^+^ macrophages. In conclusion, our results illustrated that TNVs could inhibit macrophage infiltration and polarization into M1 phenotypes, thereby regulating intestinal mucosal immunity.

### TNVs modulated the composition of gut microbiota

**Figure 6:**
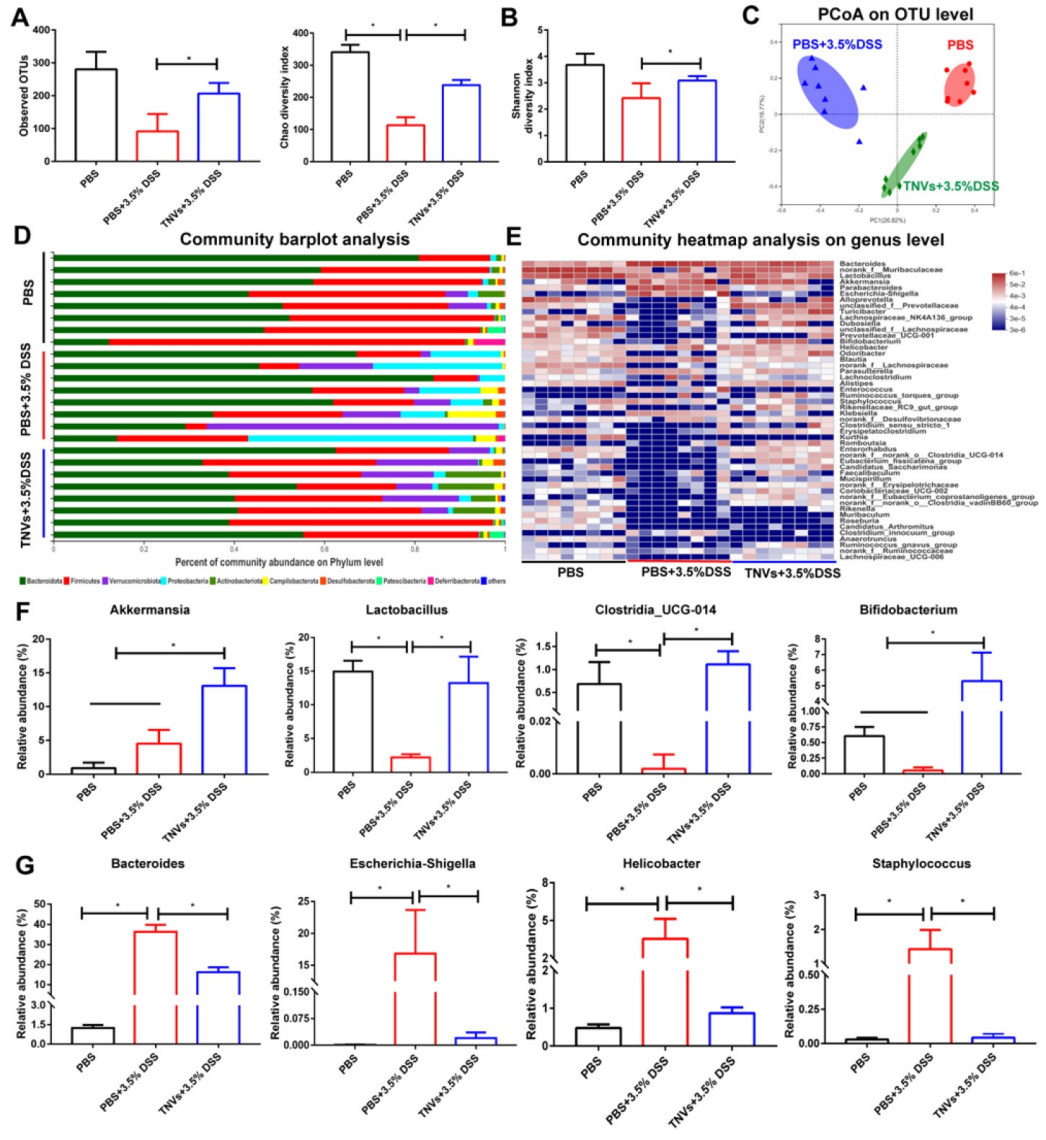
**TNVs regulated the composition of intestinal microbiota. A-B.** Estimation of microbial community observed operational taxonomic units (OTU) richness and α-diversity (chao and shannon index). **C.** PCoA plot illustrating the intestinal microbiota β-diversity.** D.** Relative abundance of intestinal microbiota. Phylum-level taxonomy are presented as a percentage of total sequences. **E**. Heatmap of the relative abundance of genus-level taxonomy for each mouse. **F-G.** Relative abundance of representative beneficial and harmful taxa. Data are shown as means ± SD; n = 8. Significance as *P < 0.05.

Mounting evidence demonstrates that gut microbiome is closely involved in the pathogenesis of UC and fecal bacteria transplantation is a strategy for UC therapy 36, 37. Therefore, whether TNVs treatment modulated the composition of gut microbiota was investigated. The results of 16S ribosomal RNA gene sequencing of fecal samples showed that TNVs treatment improved bacterial richness and diversity (Figure 6A-B). The principal co-ordinates analysis (PCoA) plots revealed that TNVs treatment group had a distinct gut microbiota profile, compared with DSS group (Figure 6C). Subsequently, the community barplot analysis showed the relative abundance of microbiota on the phylum level and the community heatmap analysis presented the relative abundance of top 50 microbiota on the genus level (Figure 6D-E). The data on genus level indicated that TNVs treatment prominently increased the relative abundance of *akkermansia*, *lactobacillus*, *clostridia_UCG-014* and *bifidobacterium* (Figure 6F), which are significantly decreased in UC patients 38. Moreover, the up-regulated relative abundances of *bacteroide*, *Escherichia-Shigella*, *helicobacter* and *staphylococcus* induced by DSS were greatly decreased after oral administration of TNVs (Figure 6G). Collectively, TNVs exerted superior therapeutic activity against colitis through modulating the intestinal microbiota.

Tight junctions (TJs) are essential to maintain the integrity of the intestinal barrier, with their expression levels found to gradually decrease during UC 39. Immunofluorescence assay revealed that TNVs treatment greatly prevented the decrease of E-cadherin, ZO-1 and occludin, compared to the DSS group (Figure S6). The similar results were also observed in HT-29 monolayer cells (Figure S5A-B). Therefore, TNVs exhibited anti-colitis activity *via* upregulating the expression of tight junction proteins and protecting the intestinal barrier function.

RNA-seq data (Figure S7A-F) demonstrated that significant change in many genes was observed and there was apparent differential expression genes (DEGs) change, regardless of healthy, model and TNVs treatment group. KEGG enrichment analysis between control and DSS group showed that the number of PI3K-Akt signaling pathway related genes was the highest, suggesting that the upstream and downstream molecules of PI3K-Akt signaling pathway have changed a lot. Base on this data, we could screen and discover anti-colitis drugs to target this pathway. According to the KEGG enrichment analysis between DSS and TNVs group, the top 3 most significant DEGs were cytokine-cytokine receptor interaction, hematopoietic cell lineage and cell adhesion molecules. The top 3 numbers were PI3K-Akt signaling pathway, cytokine-cytokine receptor interaction, and NF-κB signaling pathway. Based on these findings, the underlying anti-colitis mechanisms of TNVs might be attributed to PI3K-Akt signaling pathway, cytokine-cytokine receptor interaction, cell adhesion molecules and NF-κB signaling pathway, which provided insights to explore the function molecules in TNVs.

### TNVs alleviated colitis-related symptoms of the DSS-induced chronic colitic mice

**Figure 7:**
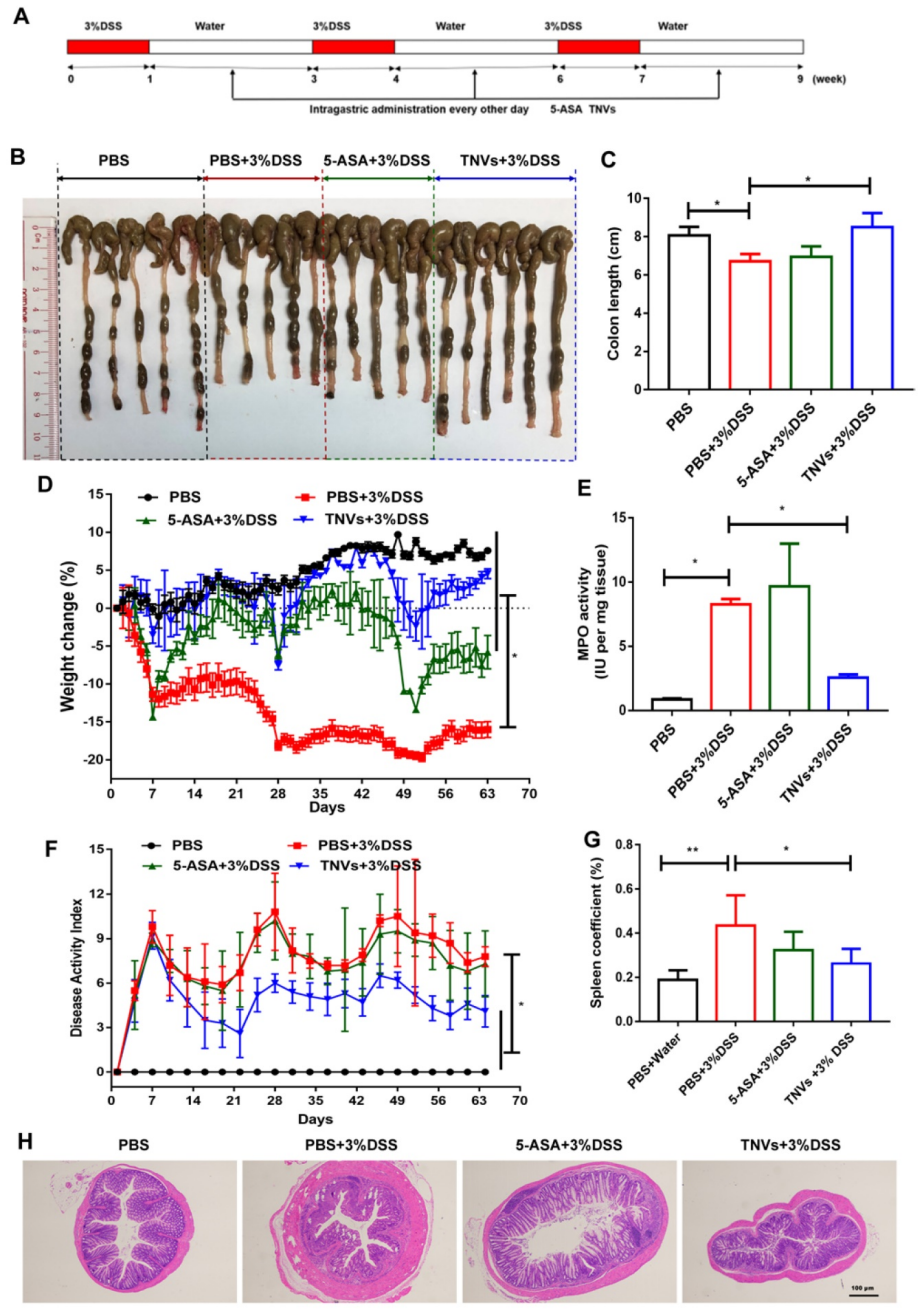
**TNVs extenuated colitis-related symptoms in DSS-induced chronic colitic mice. A.** The flow chart represents the establishment of mice colitis model and drug treatment design. **B-C**. Representative images of colons and average colon length from different groups. **D.** Body weight change expressed as the percentage of the day-zero weight. **E**. Colonic MPO activity, **F.** DAI, **G.** Spleen coefficient, and **H.** histological sections of distal colons stained with H&E (4× magnification). Data shown as means ± SD, n = 5. Significance as *P < 0.05.

The therapeutic effect of TNVs on the DSS-induced chronic colitis was further studied. In addition to healthy mice groups, other mice were randomly divided into three groups (DSS, 5-ASA + DSS, TNVs + DSS) and experienced three cycles of treatment (7 days DSS and 14 days distilled water). The scheme illustration of model establishment and drug treatment is presented in Figure 7A. The parameters, including colon length, body weight change, DAI, colonic MPO activity and pathological manifestation, were recorded and presented. Colon shortening (Figure 7B-C), body weight loss (Figure 7D) and DAI (Figure 7E) were significantly mitigated after TNVs treatment. Moreover, mice receiving TNVs greatly reduced the elevated MPO levels by DSS (Figure 7F). During chronic colitis, immune cells are activated to proliferate, and accumulate in the spleen, leading to increased spleen weight 40. The spleen coefficient was thus increased from 0.18 to 0.43 after three cycles of DSS administration (Figure 7G). Strikingly, TNVs prevented the increase of spleen weight and even recovered to the level of healthy mice, demonstrating that oral administration of TNVs could prevent DSS-associated splenomegaly. H&E staining slices of colon tissues in the experiment were examined with results supporting that TNVs treatment prominently attenuated inflammatory cell infiltration, epithelium structure disruption and loss of goblet cell (Figure 7H). Notably, mice of the 5-ASA treatment group exerted the colitis-related symptoms alleviation, the gastrointestinal side effects of long-term 5-ASA administration could not be ignored. In summary, TNVs may shed light on the prior therapeutic efficacy and translational potential for the treatment of acute and chronic colitis.

### The biosafety evaluation of TNVs in cells, zebrafish and mice

**Figure 8:**
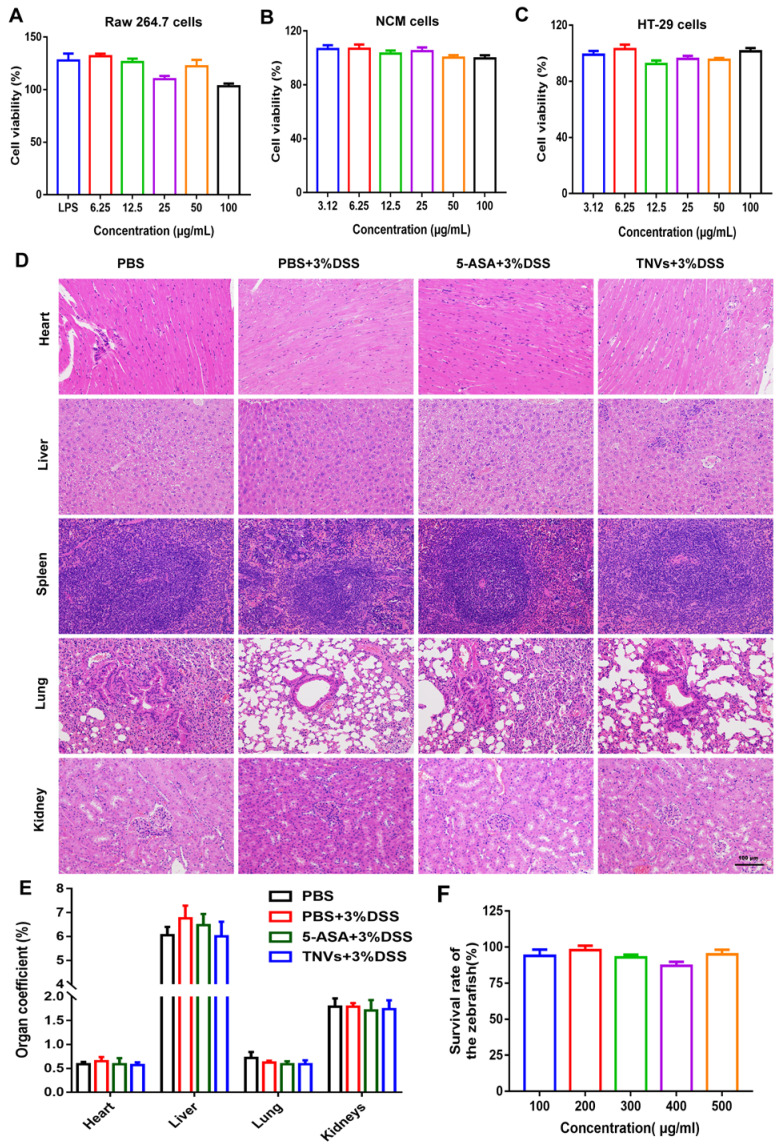
***In vitro* and *in vivo* biosafety evaluation of TNVs. A-C.** Viabilities of Raw 264.7, NCM 460 and HT-29 cells after incubation with TNVs at different concentrations for 24 h, n = 6. **D.** H&E staining of main organs slices from mice H&E staining of main organs slices from mice treated with TNVs. **E.** The organs coefficients, n = 5, and **F.** The survival rate of zebrafish after treatment with TNVs at different concentrations for 24 h, n = 15. Data shown as means ± SD.

Biosafety, as a prerequisite for the clinical translation of therapeutics, it is no exception for TNVs. Firstly, the effect of TNVs on Raw 264.7 macrophages, HT-29 cells, and NCM 460 cells (normal intestinal epithelium cells) was conducted. The cells viabilities data showed that TNVs exhibited no obvious cytotoxicity against these 3 cell lines, at concentrations up to 100 μg/mL (Figure 8A-C). Further, *in vivo* biosafety was examined by long-term oral administration of TNVs in the DSS-induced chronic colitic mice. The HE results of main organs (heart, liver, spleen, lung and kidney) in all the groups showed no apparent pathological change (Figure 8D). The organ coefficients, as a major index of chronic toxicity, were also calculated and revealed no significance in all the groups (Figure 8E). The concentration of alanine aminotransferase (ALT), aspartate aminotransferase (AST), blood urea nitrogen (BUN), creatinine (CREA) in the serum of chronic colitic mice experienced from different treatment (Figure S8) all fall into normal reference range, suggesting the good biocompatibility of TNVs. Finally, the survival rate of zebrafish was assessed with TNVs treatment at the predetermined concentration for 24 h. The data showed that TNVs did not affect the survival of zebrafish, even at a concentration of up to 500 μg/mL (Figure 8F). Overall, the above findings demonstrate that oral administered TNVs have excellent safety performance, which is appropriate for the following clinical translation.

## Discussion

UC, an idiopathic and recurrent intestinal inflammation, troubled thousands of patients and brought great burden on the economy and public health system, due to the wide prevalence and lack of potent therapeutics 41, 42. In addition to conventional therapeutic agents, novel biological drugs have been developed and tested in clinical trials 43. However, moderate efficacy and possible side effects have limited their further application 44, 45. Recently, large amounts of synthesized drug delivery systems have been exploited for UC therapy 46. Although they could accumulate in the inflamed colon sites and exhibited potential anti-inflammatory activity, the use of organic solvents, inability to produce on a large scale, and safety issues remained still formidable challenges 47. Here, TNVs were obtained from fresh turmeric, a common medicinal-food herb, with a preparation process without adding any organic solvents and could be easily produced at an industrial large scale. Therefore, TNVs may provide a simple, versatile, cheap and safe approach for UC therapy.

Emerging evidence has indicated that the gut microbiome plays a crucial role in the pathogenesis of many human diseases, including UC 48. Our work indicated that TNVs treatment could significantly improve the bacterial richness and diversity, even close to the normal mice. Decreased abundance of *lactobacillus*, *bifidobacterium* and *clostridium* have also been increased via oral administration of TNVs. However, our current studies are limited in the mice, further validation is needed to be executed in UC patients. Besides, short-chain fatty acids (SCFAs), a cluster of metabolites generated by gut microbiota, have been demonstrated to be intimately associated with a reduced risk of UC 49, 50. Future work may also focus on the metabolites of intestinal microbiota and special involved bacterial species modulated by TNVs administration.

Immune factors greatly contributed to the initiation and progression of UC. Among them, macrophages exhibit great plasticity and can differentiate into many cell subtypes. M1 macrophages exhibit pro-inflammation activity while M2 macrophages exert anti-inflammatory activity. Recent studies suggest that macrophage polarization from M1 to M2 phenotype might be harnessed to prevent the tissue injury caused by inflammation 51. Remarkably, TNVs treatment inhibited the expression of CD16/32 and elevated the expression of CD206 in the colonic lamina propria. In addition to the two well-established macrophage phenotype, more phenotypes have been sub-grouped and their mechanisms in the prevention and treatment of UC might be further studied. Otherwise, other immune cells, neutrophils, dendritic cells, T helper type (Th) 1/2/17 cells and regulatory T cells (Tregs), exert a pivotal effect in the inflammation. Whether TNVs regulated the expression and proportion of these immune cells remains to be determined.

Curcumin, one of the main active constituent extracted from dry turmeric rhizomes, exhibits multiple pharmacologic functions 52. The oral administration dose of curcumin against colitis in literatures ranged from 50 to 200 mg/kg 53. The dose of curcumin in our mice experiment was equivalent to 1.6 mg/kg, which was so low to reach therapeutic level (Table S2). Consequently, the active components in TNVs against DSS-induce colitis may be attributed to other constituents, such as proteins or RNA.

## Conclusion

In this study, TNVs were isolated, purified and characterized as a kind of uniform and nano-size spherical vesicles, which were composed of lipids, proteins, mRNA and small molecules. *In vitro* assay demonstrated that TNVs could reduce the expression of inflammatory cytokines and promote the transformation of M1 to M2. Importantly, TNVs alleviated the colitis-related symptoms through restoring the intestinal epithelium barrier, regulating the composition and relative abundance of gut microbiota and reshaping the immune microenvironment. Overall, natural TNVs with great potentials for large scale production may shed light on a safe, feasible and efficacious approach for the treatment of UC.

## Materials and Methods

### Chemicals and Reagents

Fresh turmeric was collected from Qianwei, Sichuan, China. Sucrose was purchased from Aladdin (Shanghai, China). All cell culture reagents, including fetal bovine serum (FBS), McCoy's 5a medium, Dulbecco's Modified Eagle Medium (DMEM), RPMI 1640 medium, Penicillin/Streptomycin (P/S), and 0.25% trypsin-EDTA, were purchased from Gibco. Phosphate buffered saline (PBS) tablets were obtained from Takara. 3,3ʹ-Dioctadecyloxacarbocyanine perchlorate (DiO), 1,1′-dioctadecyl-3,3,3′,3′-tetramethylindotricarbocyanine iodide (DiR) were provided from Bridgen Biotechnology Co., Ltd. Hoechst 33342, Alexa Fluor 488 and 568 was purchased from Thermo Fisher Scientific. ZO-1, occludin and E-cadherin primary antibodies were from Bioss Biotechnology Co., Ltd (Beijing, China). Recombinant murine Interleukin-4 (IL-4) and Interferon-γ (IFN-γ) were obtained from Peprotech. Dextran Sulfate Sodium Salt (DSS, MW 36-50 kDa, colitis grade) was provided by MP Biomedicals. ELISA kits (mouse TNF-α, IL-6, MCP-1) and LEGENDplex™ Mouse Inflammation Panel (13-plex) were purchased from Biolegend.

### Isolation and purification of TNVs

Fresh turmeric was washed with tap water and homogenized in a grinder. To remove the large particles of tissue and fibers, the turmeric juice was obtained through differential centrifugation following 1,000 g, 2,000 g, 3,000 g, and 10,000 g for 10, 20, 30, and 60 min, respectively. Subsequently, the above obtained juice was centrifuged at 150,000 g for 1.5 h, with the pellet ultrasonically resuspended and dispersed in PBS. For the purification of TNVs, the suspension was transferred to a discontinuously dispersed sucrose gradient (8%, 30%, 45%, and 60%) solutions, and centrifuged at 150,000 g for 1.5 h. Three kinds of TNVs from different bands were harvested. The protein concentrations of the obtained TNVs were determined by using a BCA protein assay kit and stored at -80°C for further use.

### Characterization of TNVs

For the characterization of TNVs, the size distribution, polydispersity index (PDI) and zeta potential were determined by DLS (Malvern, Worcestershire, WR, UK). To observe the morphology of the TNVs, the corresponding samples were stained with 1% (w/v) uranyl acetate and imaged under TEM (JEM-2100F, Tokyo, Japan).

### Compositional analysis of TNVs

#### Lipidomic analysis

TNVs were subjected to lipidomic analysis using ultra high-performance liquid chromatography tandem time-of-flight mass spectrometry system (UPLC/TOF-MS). The samples were separated on a HSS T3 column (150 mm × 2.1 mm, 3 µm; Waters, Milford, USA); 2 mM ammonium formate and 0.05% formic acid in water (A), and acetonitrile/isopropanol (1:1, v/v) (B) were the mobile phases. The gradient program was: 0-2 min, 2% B; 2-7 min, 2-70% B; 7-14 min, 70-90% B; 14-16 min, 90-100% B; 16-20 min, 100% B. 3 μL sample was injected and run at a flow rate of 0.30 mL/min. The column temperature was set at 40°C. The sample mass spectrum signal acquisition adopted positive ion (ESI+) and negative ion (ESI-) scanning modes. The electrospray capillary voltages were set at 5500 V (ESI+) and -4500 V (ESI-), TOF-MS scan range was 70-1000 m/z and ion source temperature were set to 550℃.

#### Proteomic analysis

TNVs were shipped to Shanghai Omicsolution Co., Ltd. in dry ice. Proteins in TNVs were analyzed by LC-MS/MS. The sample was separated on an Acclaim PepMap C18 column. The elution program was: 0-60 min, the rate of mobile phase B (0.1% formic acid in acetonitrile) ranging from 4% to 50%. The flow rate was 0.3 mL/min and the column temperature was 40°C. The main parameters of MS and MS/MS modes were as follows: (1) MS: scan range (m/z), 350-1600; resolution 60,000; (2) HCD-MS/MS: resolution 150,000. Finally, PEAKS Studio version X was applied to process the mass spectra.

#### Curcuminoid content determination in TNVs

To extract curcuminoids, TNVs were mixed well with 9 times volume of methanol and then centrifuged to obtain the supernatant for LC-MS analysis. The samples were separated using a Hypersil gold C18 (Thermo Fisher) column with column temperature at 40°C. The mobile phase consisted of 0.1% formic acid (v/v) in water (A) and acetonitrile (B). The gradient program was: 0-2 min, 5% B; 2-25 min, 5-100% B; 25-30 min, 100% B. The flow rate was 0.4 mL/min and the injection volume was 2 μL. For the ESI-HRMS analysis, positive ESI ion mode was used to analyzed the samples. The main parameters of ESI were as follows: ion spray voltage, 4.5 kV; capillary temperature, 380°C; capillary voltage, 35 V. All quantitative data in this study were acquired using full MS scan mode.

### The stability of TNVs in the simulated gastric and intestinal fluids

Simulated gastric fluids (SGF) and simulated intestinal fluids (SIF) were prepared according to USP. The formula of SGF and SIF were 80 mM HCl, 35mM NaCl, 0.3% pepsin, pH 1.2 and 15 mM NaOH, 50 mM KH2PO4, 1% pancreatin, pH 6.8, respectively. TNVs were disperse in SGF and SIF at a concentration of 0.1 mg/mL. After incubation at 200 rpm, 37°C for 2 h, the particle size of TNVs in both fluids were measured by DLS.

### Animals and cell lines

ICR mice (female and male, 6-8 weeks old) were purchased from the Faculty of Health Sciences of the University of Macau. All animal studies were conducted following the NIH Guidelines for the Care and Use of Laboratory Animals, and the animal experimental protocols were approved by the University of Macau Animal Ethics Committee.

Raw 264.7 murine macrophage, NCM 460 and HT-29 colonic epithelial cell were obtained from the American Type Culture Collection. Raw 264.7 cells were cultured in DMEM medium containing 10% (v/v) FBS. NCM 460 cells and HT-29 cells were cultured in RPMI 1640 medium and McCoy's 5a medium, both containing 10% (v/v) FBS and 100 IU/mL penicillin/streptomycin. These above cells were all cultured in a humidified 5% CO_2_ at 37°C.

### *In vitro* cytotoxicity evaluation

To assess the cytotoxicity of TNVs on the Raw 264.7, NCM 460 and HT-29 cells, 3-(4,5-dimethylthiazol-2-yl)-2,5-diphenyltetrazolium bromide (MTT) assays were conducted. Firstly, cells were seeded in a 96-wells plate with a density of 1 × 10^4^ cells/well and incubated overnight to adherent. Subsequently, TNVs were diluted into different concentrations with the corresponding complete medium and added to the wells. After incubation for 24 h, the TNVs-contained mediums were replaced with 0.5 mg/mL MTT solution. 4 h later, the MTT solutions were discarded and then 100 μL DMSO were added to the wells to dissolve the precipitation. After shaken for 10 min, the plates were scanned via a FlexStation 3 multi-mode microplate reader at 490 and 570 nm.

### Cellular uptake and the endocytosis mechanism assays

To observe the cellular uptake of DiO labeled TNVs in NCM 460 and Raw 264.7 cells. Both cells were seeded at a density of 2 × 10^5^ cells/well in 24-well plate and incubated with DiO labeled TNVs for 1, 3, and 6 h. Subsequently, the cells were digested or scraped off to form single-cell suspension, and then wash for three times with cold PBS. Finally, the cell samples were analyzed by flow cytometer (Beckman Cytoflex, USA). In addition, both cells were seeded in confocal dishes, incubated with DiO labeled TNVs, washed with PBS and stained with Hoechst 33342 for nuclear localization. The cellular uptakes of TNVs were imaged by Leica TCS SP8 microscope.

To investigate the endocytosis mechanism of TNVs in Raw 264.7 and NCM 460 cells, multiple endocytosis inhibitors were used. Compared with the above cellular take assay, both cells were pre-treated with various inhibitors for 1 h. The inhibitors and concentrations were as follows: 5 μM methyl-β-cyclodextrin (M-CD), 5 μg/mL filipin, 10 μg/mL chlorpromazine (CLP), 2 mM sodium azide (NaN3), 3 mM 5-(N, N-dimethyl) amiloride hydrochloride (AMH), and 200 μM genistein. In addition, the effect of low temperature on TNVs uptake by both cells was also studied. The subsequent cell treatment procedure was similar to the cellular uptake assay. Finally, the cells were analyzed using flow cytometer and captured by Leica TCS SP8 microscope.

### Anti-inflammatory activity of TNVs *in vitro*

To study the anti- inflammatory activity of TNVs, Raw 264.7 cells were seeded at a density of 1 × 10^5^ cells/well in 24-well plates and incubated with TNVs (25 μg/mL), LPS (1 μg/mL) or blank medium. After incubation for 24 h, the supernatants were harvested, and the levels of various pro-inflammatory factors (TNF-α, IL-6, MCP-1) were determined by using corresponding enzyme-linked immunosorbent assay (ELISA) kits according to the manufacturer's instructions.

### The effect of TNVs on macrophage polarization

Raw 264.7 cells were seeded on 6-well plates at a density of 2 × 10^6^ cells/well. 100 ng/mL LPS and 20 ng/mL IFN-γ were used to treat cells for up to 48 h to generate M1 macrophages. Similarly, 20 ng/mL IL-4 was used to stimulate cells to generate M2 macrophages. To identify the effect of TNVs on the macrophage polarization, TNVs were co-treated with M1 or M2 inducer. Finally, the cells were harvested, blocked and stained with antibodies (surface markers such as F4/80, CD11b, CD206) for the flow cytometer analysis.

### The immunofluorescence staining of tight junction proteins in HT-29 monolayer cells

HT-29 cells were seeded in the confocal dish at a density of 2.0 × 10^4^, and then cultured in 5% CO_2_, 37°C incubator. The culture mediums were replaced every other day and the cell monolayer was formed after 10 days. Subsequently, the monolayer was treated with TNVs and then fixed, stained, captured by confocal microscope to observe the expression level of tight junction proteins.

### The therapeutic efficacy of TNVs on the DSS-induced acute colitic mice

Before the experiment, 6-8 weeks old ICR mice were housed and acclimatized for 7 days. 3.5% DSS dissolved in the drinking water were given to mice for 12 days. Afterwards, 10 mg/kg TNVs, 100 mg/kg 5-ASA or PBS was orally administered into mice every day and control mice were only given to normal drinking water. The colitis-related symptoms, including stool consistency, body weight and fecal bleeding, were monitored and recorded daily during the experimental period. On the 12^th^ day, mice were sacrificed and the entire colon was harvested. Feces were collected for microbiome analysis. 1.0 cm distal colons were fixed in 4% paraformaldehyde and used for histological assessment and immunofluorescence staining. Other colon tissues were used for flow cytometry analysis and the measurement of MPO activity and cytokines level.

### Isolation of lamina propria cells and FACS analysis of macrophage population

After the animal experiment, the cells were isolated from mice colonic lamina propria suffering from different treatment according to a previous report 54, 55. In brief, the colons were exercised, cut along the intestinal axis, washed with cold PBS twice and cut into small pieces. Then the small colon pieces were incubated in Ca/Mg-free HBSS containing 2 mM EDTA three times at 37°C for 10 min under shaking. After that, the tissues were digested with RPMI medium containing 20% FBS, 1 mg/mL collagenase A and 0.05 mg/mL DNAase at 37°C for 10 min under 200 rpm shaking. Subsequently, the digestion process was stopped; the suspensions were centrifugated and filtered through 100 μm and 40 μm cell strainers to obtain the mononuclear cell suspensions. Finally, the mononuclear cell suspensions were blocked and stained with antibodies (surface markers such as CD11b, F4/80, CD16/32, CD206) for the flow cytometer analysis.

### Microbiome analysis

Mouse feces samples were shipped on dry ice to Majorbio Bio-Pharm Technology Co. Ltd. for microbiome analysis. Briefly, genomic DNA was extracted by using the E.Z.N.A.® soil DNA Kit. Then, the extracted DNA was separated on agarose gel, and NanoDrop 2000 UV-vis spectrophotometer was applied to determine DNA concentration and purity. Primer pairs 338F and 806R were used to amplify the hypervariable region V3-V4 of the bacterial 16S rRNA gene by an ABI GeneAmp® 9700 PCR thermocycler. Purified amplicons were sequenced on the Illumina MiSeq sequencing platform using PE300 chemical. The related analysis of the 16S rRNA microbiome sequencing data was performed using Majorbio Cloud Platform (www.majorbio.com).

### The therapeutic efficacy of TNVs on DSS-induced chronic colitic mice

The chronic colitis model induced by DSS was established according to a previous method 56. The mice would suffer from 3-cycle DSS treatment to induce chronic colitis, each containing 1-week 3% DSS followed by 2-week drinking water. The mice were divided into 4 groups: control group (without any treatment); model group, 5-ASA group and TNVs treatment group. 5-ASA and TNVs were orally administered at each interval of the cycle every other day. During the experiment period, the body weight, stool consistency and fecal bleeding were monitored and recorded. Different from the acute colitis, the main organs (heart, liver, spleen, lung and kidney) were excised, weighed and fixed for histological examination. The levels of ALT, AST, BUN and CREA in the serum were also measured by automatic biochemical analyzer.

### Biodistribution study of TNVs

Oral administration of DiR-labeled TNVs was given to the DSS-induced acute colitic mice. Subsequently, the distribution of TNVs was captured and imaged by the IVIS imaging system (Caliper PerkinElmer, Hopkinton, USA) at the predetermined time point. After that, the corresponding mice were sacrificed and their colons were excised to *ex-vivo* imaging. To furtherly study the therapeutic effect of TNVs, the mice experienced drug treatment was also given DiR-labeled TNVs to observe the distribution of fluorescence intensity in the colons.

### Colonic MPO activity assay

The above harvested colons were washed with cold PBS and then homogenized in cold 50 mM potassium phosphate buffer containing 0.5% hexadecyltrimethylammonium bromide. MPO in the supernatant was detected by adding 0.167 mg/mL of o-dianisidine dihydrochloride and 0.005% H_2_O_2_. The changes in absorbance at 450 nm were recorded over 5 min 57.

### Immunofluorescence staining

For immunofluorescence staining, the cells and frozen colon slices were fixed in 4% paraformaldehyde, permeabilized with 0.1% Triton X-100, blocked non-specific proteins in 1% BSA/PBS for 1 h, and incubated 12 h at 4°C with ZO-1, Occludin and E-cadherin primary antibodies at the proper dilution times. The confocal dishes and slides were then washed with PBS and incubated with Alexa Fluor 488 and/or Alexa Fluor 568 and Hoechst 33342 overnight at 4°C. Finally, the fluorescence staining cell monolayer and colon slices were imaged and captured using Leica TCS SP8 microscope.

### Histological staining

Mice colons and other main organs were fixed in 4% paraformaldehyde, then embedded in paraffin, sectioned at 6 μm thickness and stained with H&E. Images were captured using a Nikon Eclipse Ci Biological Microscope with DS-RI2 Camera.

### Statistical analysis

Statistical analysis was performed by using the GraphPad Prism 7.0 software. Statistical differences were determined by unpaired Student's t-test or one‐way ANOVA with a post hoc Bonferroni test. Data were presented as mean ± SD. *P* value <0.05 was considered statistically significant, and ns means no significance.

## Supplementary Material

Supplementary figures and tables.

## Figures and Tables

**Scheme 1:**
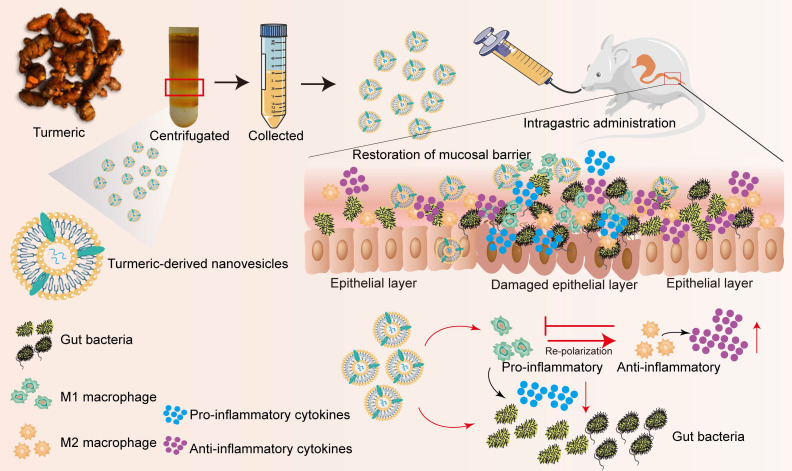
** Schematic illustration of the preparation procedure and anti-colitic efficacy of TNVs**. TNVs exert potent anti-inflammation activity via restoring the damaged intestinal barrier, modulating the intestinal microbiota and altering the aberrant mucosa immune environment, especially reshaping the macrophage polarization condition.

## Abbreviations

5-ASA: 5-aminosalicylic acid
AMH: amiloride hydrochloride
CD: cluster of differentiation
CLP: chlorpromazine
DSS: dextran sulfate sodium
DLS: dynamic light scattering
DAG: diacylglycerols
DiO: 3,3'-dioctadecyloxacarbocyanine
DiR: 1,1'-dioctadecyl-3,3,3',3'-tetramethylindotricarbocyanine iodide
DAI: disease activity index
DMEM: dulbecco's modified eagle medium
ELISA: enzyme-linked immunosorbent assay
FACS: fluorescence-activated cell sorting
FA: fatty acids
FBS: fetal bovine serum
GO: Gene Ontology
HE: hematoxylin-eosin
IBD: inflammatory bowel disease
IL-6: Interleukin 6
KEGG: Kyoto Encyclopedia of Genes and Genomes
LPS: lipopolysaccharide
LC-MS: liquid chromatography-mass spectrometry
MTT: 3-(4,5-dimethylthiazol-2-yl)-2,5-diphenyltetrazolium bromide
M-β-CD: methyl-beta-cyclodextrin
MCP-1: monocyte chemoattractant protein 1
MPO: myeloperoxidase
OTU: operational taxonomic units
PC: phosphatidylcholine
PE: phosphatidyl ethanolamine
PCoA: principal co-ordinates analysis
P/S: penicillin/streptomycin
PBS: phosphate buffered saline
PDI: polydispersity index
SCFAs: short-chain fatty acids
SGF: simulated gastric fluids
SIF: simulated intestinal fluids
TNF: tumor necrosis factor
TEM: transmission electron microscope
TAG: triacylglycerols
TJs: tight junctions
Th: T helper type
Tregs: regulatory T cells
UC: ulcerative colitis
UPLC/TOF-MS: ultra-high-performance liquid chromatography tandem time-of-flight mass spectrometry

## References

[1] Xavier RJ, Podolsky DK (2007). Unravelling the pathogenesis of inflammatory bowel disease. Nature.

[2] Kaplan GG, Ng SC (2017). Understanding and preventing the global increase of inflammatory bowel disease. Gastroenterology.

[3] Hanauer SB (2004). Update on the etiology, pathogenesis and diagnosis of ulcerative colitis. Nat Clin Pract Gastroenterol Hepatol.

[4] Neurath MF (2017). Current and emerging therapeutic targets for IBD. Nat Rev Gastroenterol Hepatol.

[5] Dubinsky MC (2004). Azathioprine, 6-mercaptopurine in inflammatory bowel disease: pharmacology, efficacy, and safety. Clin Exp Gastroenterol Hepatol.

[6] Hoentjen F, Van Bodegraven AA (2009). Safety of anti-tumor necrosis factor therapy in inflammatory bowel disease. World J Gastroenterol.

[7] Singh S, Murad MH (2020). First-and second-line pharmacotherapies for patients with moderate to severely active ulcerative colitis: an updated network meta-analysis. Clin Exp Gastroenterol Hepatol.

[8] Lautenschläger C (2014). Drug delivery strategies in the therapy of inflammatory bowel disease. Adv Drug Deliv Rev.

[9] Zhang S (2017). Nanoparticulate drug delivery systems targeting inflammation for treatment of inflammatory bowel disease. Nano Today.

[10] Hassan S (2017). Evolution and clinical translation of drug delivery nanomaterials. Nano today.

[11] Andaloussi SE (2013). Extracellular vesicles: biology and emerging therapeutic opportunities. Nat Rev Drug Discov.

[12] Van Niel G (2018). Shedding light on the cell biology of extracellular vesicles. Nat Rev Mol Cell Biol.

[13] Buzas EI (2014). Emerging role of extracellular vesicles in inflammatory diseases. Nat Rev Rheumatol.

[14] Thery C (2015). Diagnosis by extracellular vesicles. Nature.

[15] Vader P (2016). Extracellular vesicles for drug delivery. Adv Drug Deliv Rev.

[16] He C (2018). Exosome theranostics: biology and translational medicine. Theranostics.

[17] Wang Q (2015). Grapefruit-derived nanovectors use an activated leukocyte trafficking pathway to deliver therapeutic agents to inflammatory tumor sites. Cancer Res.

[18] Zhang M (2016). Edible ginger-derived nanoparticles: A novel therapeutic approach for the prevention and treatment of inflammatory bowel disease and colitis-associated cancer. Biomaterials.

[19] Deng Z (2017). Broccoli-derived nanoparticle inhibits mouse colitis by activating dendritic cell AMP-activated protein kinase. Mol Ther.

[20] Teng Y (2018). Plant-derived exosomal microRNAs shape the gut microbiota. Cell Host Microbe.

[21] Cao M (2019). Ginseng-derived nanoparticles alter macrophage polarization to inhibit melanoma growth. J Immunother Cancer.

[22] Liu B (2021). Therapeutic potential of garlic chive-derived vesicle-like nanoparticles in NLRP3 inflammasome-mediated inflammatory diseases. Theranostics.

[23] Sundaram K (2022). Garlic exosome-like nanoparticles reverse high-fat diet induced obesity via the gut/brain axis. Theranostics.

[24] Henning SM (2011). Antioxidant capacity and phytochemical content of herbs and spices in dry, fresh and blended herb paste form. Int J Food Sci Nutr.

[25] Díaz-Maroto MC (2003). Influence of drying on the flavor quality of spearmint (Mentha spicata L.). J Agric Food Chem.

[26] Xu X (2021). Plant exosomes as novel nanoplatforms for microRNA transfer stimulate neural differentiation of stem cells in vitro and in vivo. Nano Lett.

[27] Gupta SC (2013). Therapeutic roles of curcumin: lessons learned from clinical trials. AAPS J.

[28] Singla V (2014). Induction with NCB-02 (curcumin) enema for mild-to-moderate distal ulcerative colitis - A randomized, placebo-controlled, pilot study. J Crohns Colitis.

[29] Neurath MF (2014). Cytokines in inflammatory bowel disease. Nat Rev Immunol.

[30] Ruytinx P (1930). Chemokine-induced macrophage polarization in inflammatory conditions. Front Immunol. 2018.

[31] Kinouchi Y (1998). Telomere shortening in the colonic mucosa of patients with ulcerative colitis. J Gastroenterol.

[32] Bennebroek Evertsz' F (2013). The patient simple clinical colitis activity index (P-SCCAI) can detect ulcerative colitis (UC) disease activity in remission: a comparison of the P-SCCAI with clinician-based SCCAI and biological markers. J Crohns Colitis.

[33] McConnico R (1999). Myeloperoxidase activity of the large intestine in an equine model of acute colitis. Am J Vet Res.

[34] Wang Z (2015). Oxidative stress and carbonyl lesions in ulcerative colitis and associated colorectal cancer. Oxid Med Cell Longev.

[35] Seyedizade SS (2020). Current status of M1 and M2 macrophages pathway as drug targets for inflammatory bowel disease. Arch Immunol Ther Exp.

[36] Ryan FJ (2020). Colonic microbiota is associated with inflammation and host epigenomic alterations in inflammatory bowel disease. Nat Commun.

[37] Schirmer M (2019). Microbial genes and pathways in inflammatory bowel disease. Nat Rev Microbiol.

[38] Manichanh C (2012). The gut microbiota in IBD. Nat Rev Gastroenterol Hepatol.

[39] Landy J (2016). Tight junctions in inflammatory bowel diseases and inflammatory bowel disease associated colorectal cancer. World J Gastroenterol.

[40] Hall LJ (2011). Induction and activation of adaptive immune populations during acute and chronic phases of a murine model of experimental colitis. Dig Dis Sci.

[41] Ananthakrishnan AN (2015). Epidemiology and risk factors for IBD. Nat Rev Gastroenterol Hepatol.

[42] Kaplan GG (2015). The global burden of IBD: from 2015 to 2025. Nat Rev Gastroenterol Hepatol.

[43] Chudy-Onwugaje KO (2019). A state-of-the-art review of new and emerging therapies for the treatment of IBD. Inflamm Bowel Dis.

[44] Hisamatsu T (2019). Concerns and side effects of azathioprine during adalimumab induction and maintenance therapy for Japanese patients with Crohn's Disease: a subanalysis of a prospective randomised clinical trial [DIAMOND Study]. J Crohns Colitis.

[45] Martinez B (2017). Patient understanding of the risks and benefits of biologic therapies in inflammatory bowel disease: insights from a large-scale analysis of social media platforms. Inflamm Bowel Dis.

[46] Liu P (2021). Receptor-mediated targeted drug delivery systems for treatment of inflammatory bowel disease: opportunities and emerging strategies. Acta Pharm Sin B.

[47] Sindhwani S, Chan WC (2021). Nanotechnology for modern medicine: next step towards clinical translation. J Intern Med.

[48] Nishida A (2018). Gut microbiota in the pathogenesis of inflammatory bowel disease. Clin Exp Hepatol.

[49] Parada Venegas D (2019). Short chain fatty acids (SCFAs)-mediated gut epithelial and immune regulation and its relevance for inflammatory bowel diseases. Front Immunol.

[50] Li C (2019). A proresolving peptide nanotherapy for site-specific treatment of inflammatory bowel disease by regulating proinflammatory microenvironment and gut microbiota. Adv Sci.

[51] Murray PJ (2017). Macrophage polarization. Annu Rev Physiol.

[52] Patel SS (2020). Cellular and molecular mechanisms of curcumin in prevention and treatment of disease. Crit Rev Food Sci Nutr.

[53] Gong Z (2018). Curcumin alleviates DSS-induced colitis via inhibiting NLRP3 inflammsome activation and IL-1β production. Mol Immunol.

[54] Jørgensen PB (2021). Identification, isolation and analysis of human gut-associated lymphoid tissues. Nat Protoc.

[55] Zhou Y (2022). Rhein regulates redox-mediated activation of NLRP3 inflammasomes in intestinal inflammation through macrophage-activated crosstalk. Br J Pharmacol.

[56] Wirtz S (2017). Chemically induced mouse models of acute and chronic intestinal inflammation. Nat Protoc.

[57] Wu M (2021). PI3KC3 complex subunit NRBF2 is required for apoptotic cell clearance to restrict intestinal inflammation. Autophagy.

