# Supplementary material for: Turmeric-derived nanovesicles as novel nanobiologics for targeted therapy of ulcerative colitis
Source: Theranostics. 2022 Jul 18;12(12):5596–614. doi: 10.7150/thno.73650 (PMC9330521; doi:10.7150/thno.73650)
Supplement: Supplementary file 1 — Supplementary figures and tables. [file thnov12p5596s1.pdf]

Supporting Information

Turmeric-derived nanovesicles as novel nanobiologics for targeted therapy of ulcerative colitis

Caifang Gao[1], Yangyang Zhou[1], Zhejie Chen[1], Hongyi Li[1], Yaqin Xiao[1], Wei Hao[1], Ying Zhu[2], Chi Teng Vong[1], Mohamed A. Farag[3], Yitao Wang[1,*] , Shengpeng Wang[1,*]

[1] State Key Laboratory of Quality Research in Chinese Medicine, Institute of Chinese Medical Sciences, University of Macau, Avenida da Universidade, Macau, China

[2] Department of Integrative Oncology, Fudan University, Shanghai Cancer Center, Shanghai 200032, China

[3] Pharmacognosy Department, College of Pharmacy, Cairo University, Kasr el Aini St., Cairo 11562, Egypt

***Correspondence:**

Yitao Wang, Institute of Chinese Medical Sciences, University of Macau, Avenida da Universidade, Taipa, Macau 999078, China. Email: YTWang@um.edu.mo

Shengpeng Wang, Institute of Chinese Medical Sciences, University of Macau, Avenida da Universidade, Taipa, Macau 999078, China. Email: SWang@um.edu.mo

Figure S1. The lipid profiles and protein composition in TNVs. A. the lipid profiles of TNVs under positive and negative mode; **B-C.** the GO and KEGG classification of TNVs.

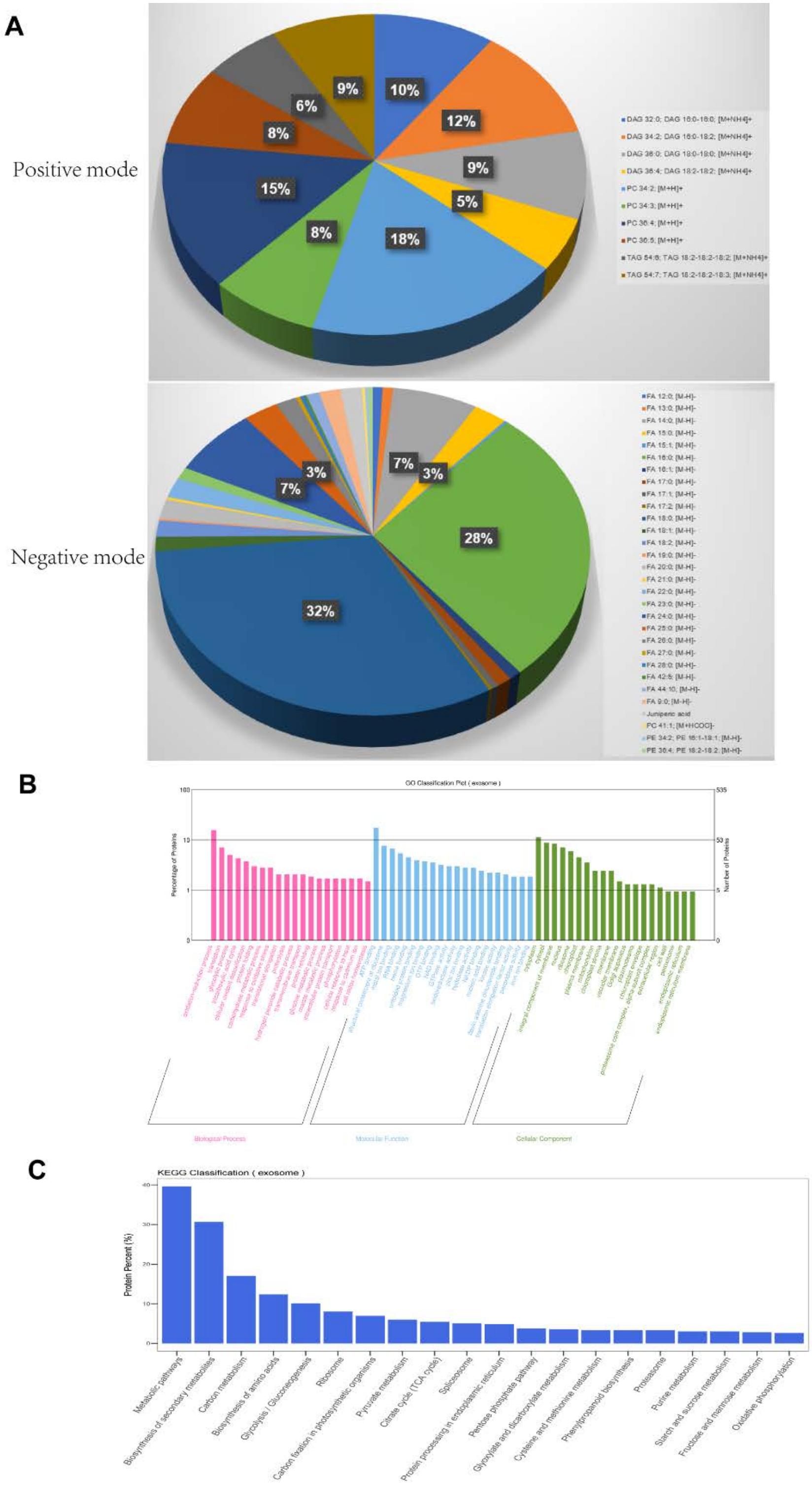

Figure S2. The size and poly dispersion index (PDI) of TNVs in PBS, stimulated gastric and intestinal fluids.

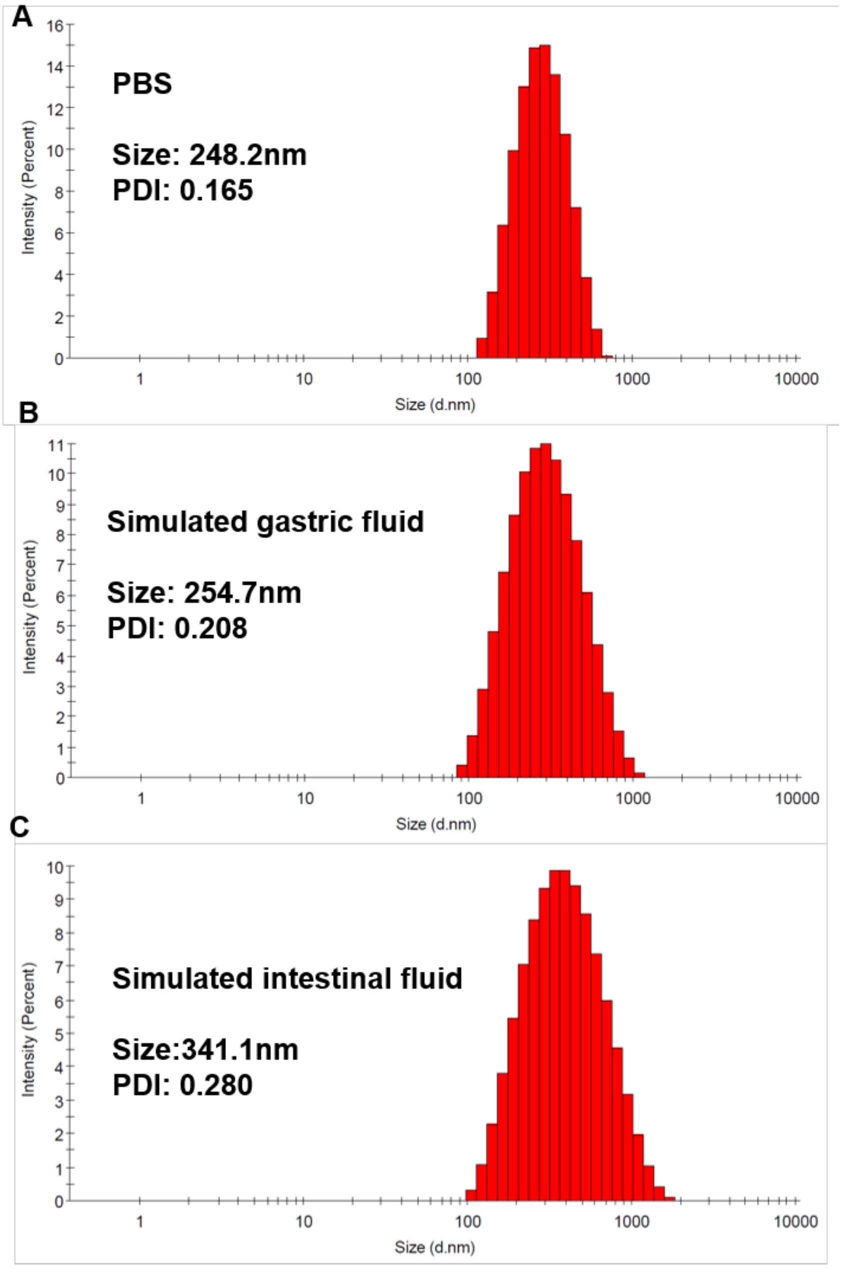

Figure S3. The cellular uptake of DiO-labeled TNVs in NCM 460 and Raw 264.7 cells for 1, 3. 6 h. A. the confocal images in both NCM 460 and Raw 264.7 cells; **B-C.** the flow cytometer result and statistic data in both cells.

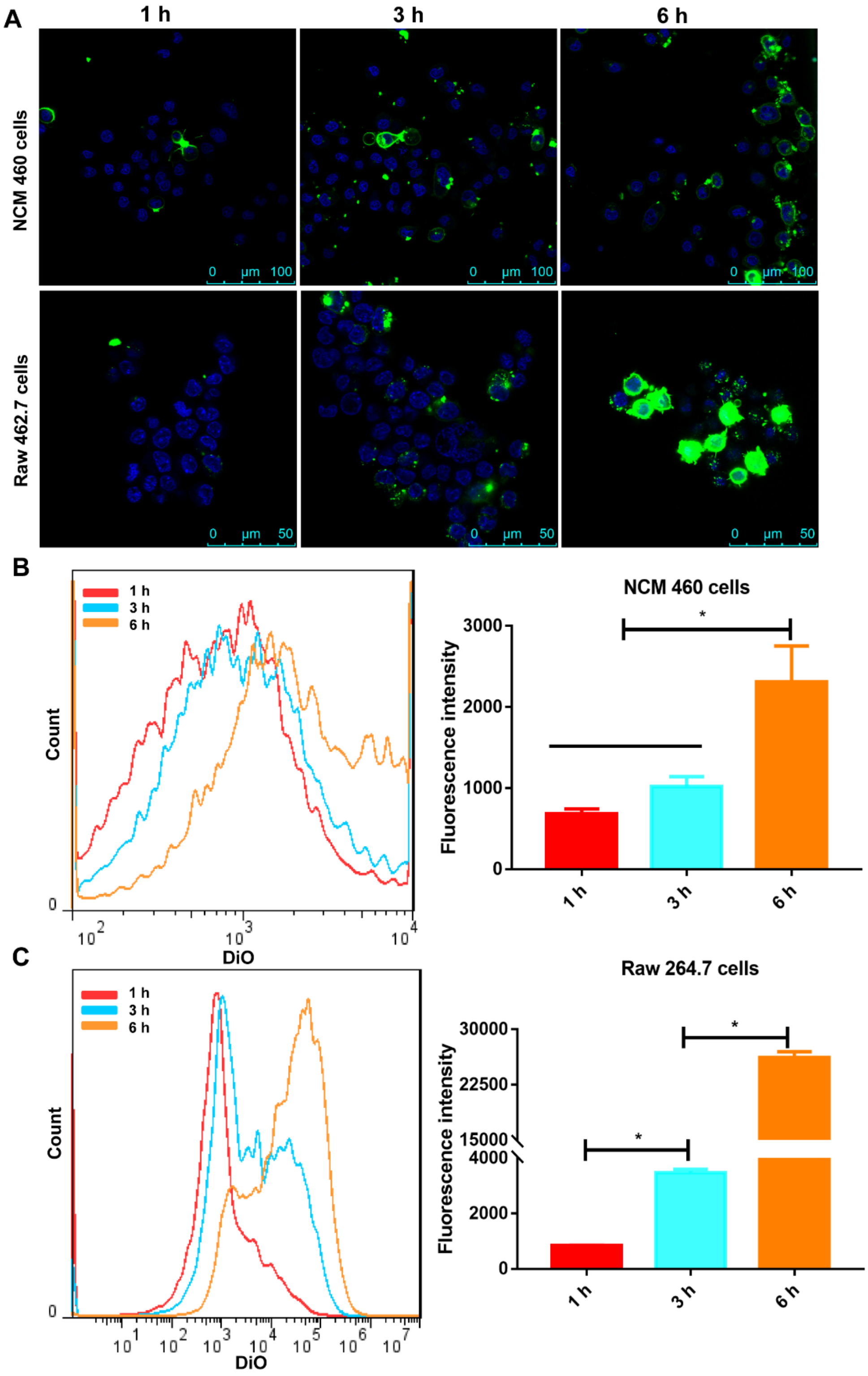

Figure S4. The fluorescence picture and fluorescence intensity of TNVs uptake in Raw 264.7 cells with LPS-stimulated condition or not, scale bar: 20μm.

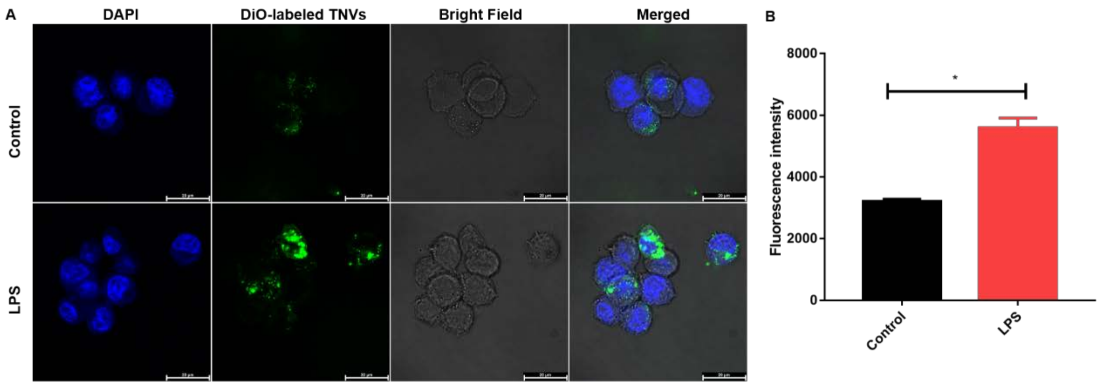

Figure S5. The *in-vitro* immunostaining of A. E-cadherin (red) and B. ZO-1 (green). HT-29 cells were cultured for 10 days into monolayer, and then treated with TNVs for 18 h, respectively. Nuclei were stained with Hoechst 33342 (blue).

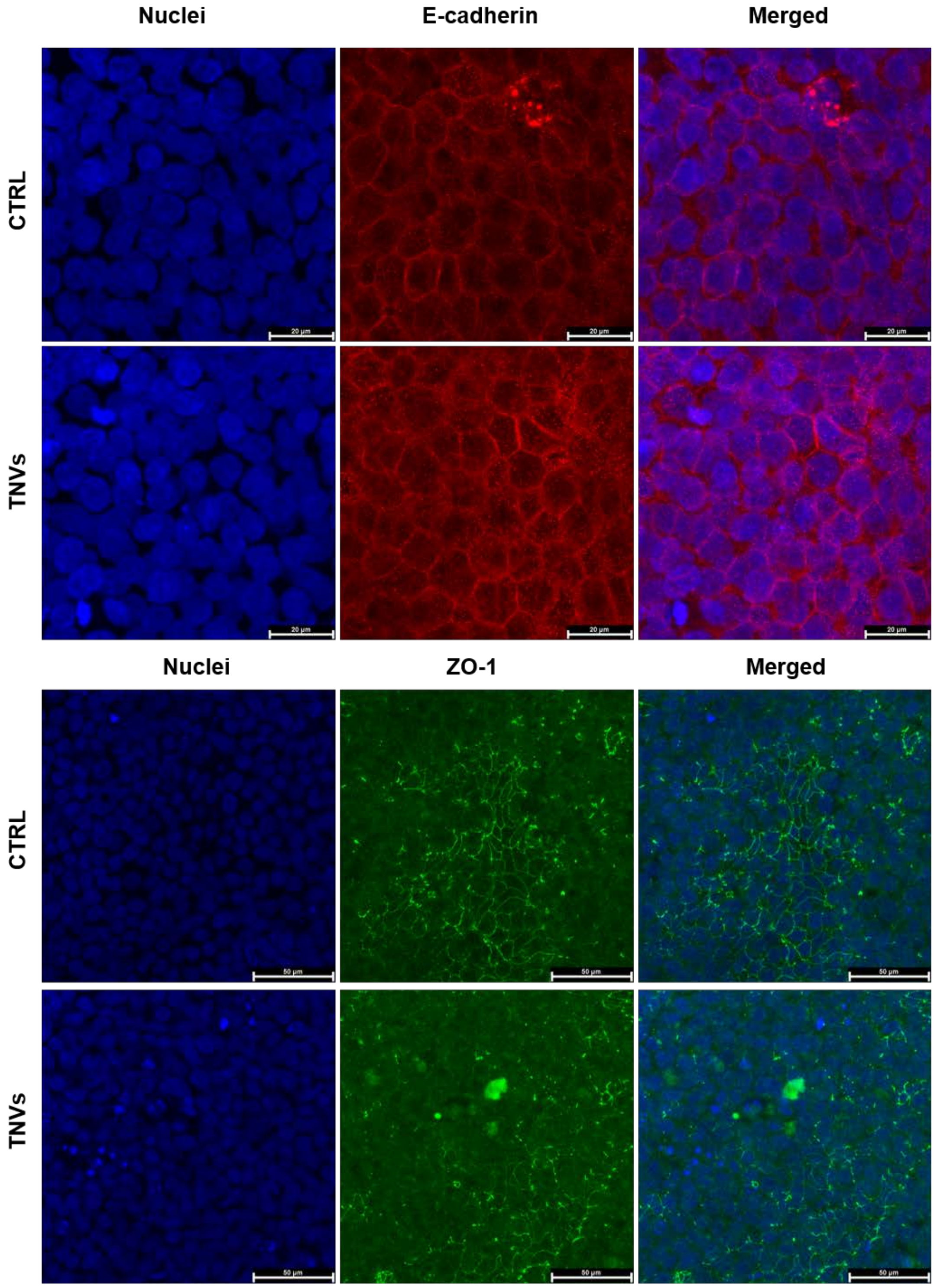

Figure S6. The *in-vitro* immunostaining of E-cadherin, ZO-1 and Occludin. Colons from acute colitis mice undergoing different treatment. Nuclei were stained with Hoechst 33342 (blue).

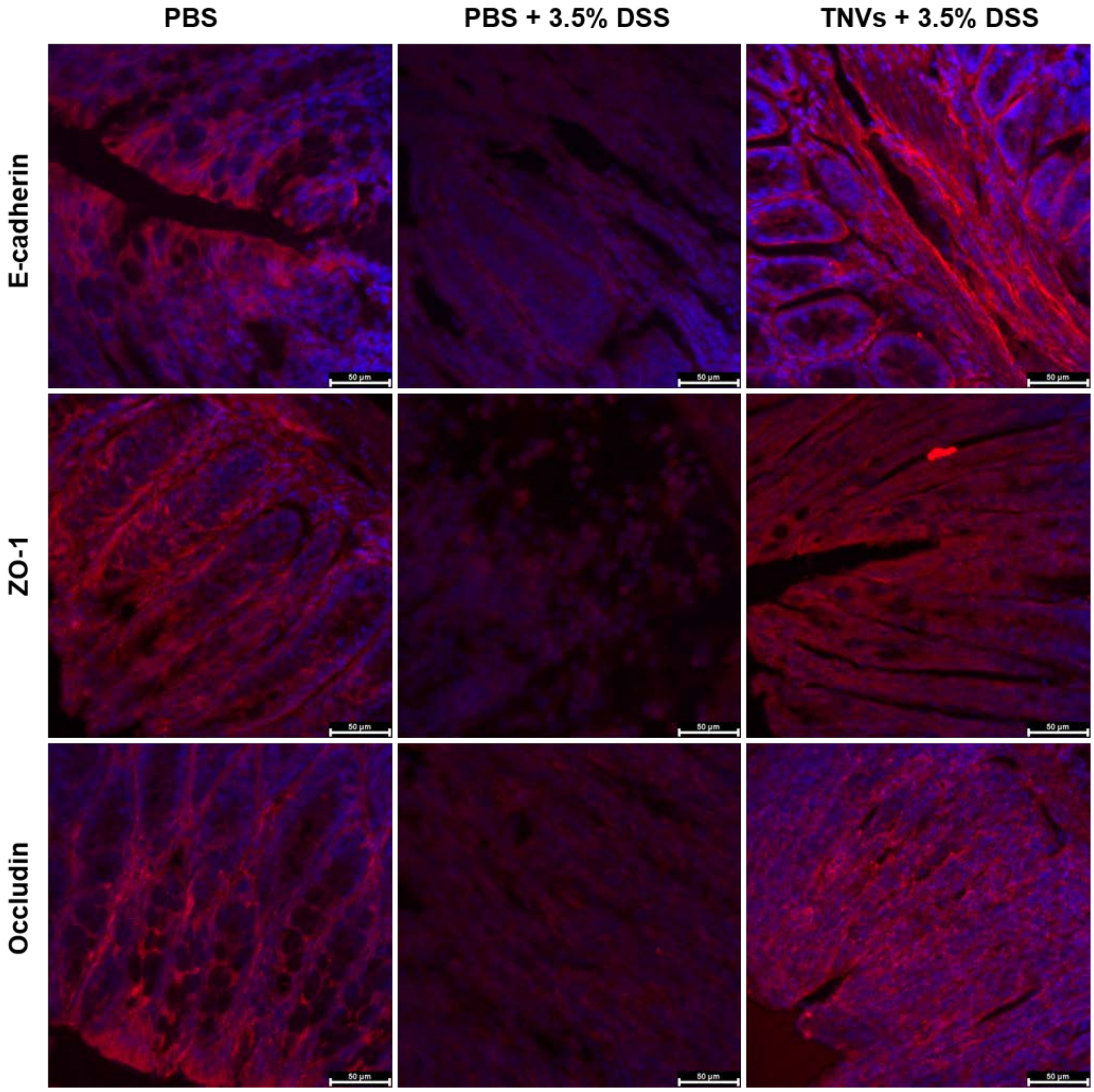

Figure S7. Colon RNA sequence and DEG from different treatment group. A. Heatmap showing the gene expression correlation of all sequenced samples; **B**. Bar plot showing the numbers of up- and downregulated DEG in the 3 groups; **C. E.** the volcano of CTRL vs. DSS and DSS vs TNVs; **D. F.** the KEGG enrichment analysis of CTRL vs. DSS and DSS vs TNVs.

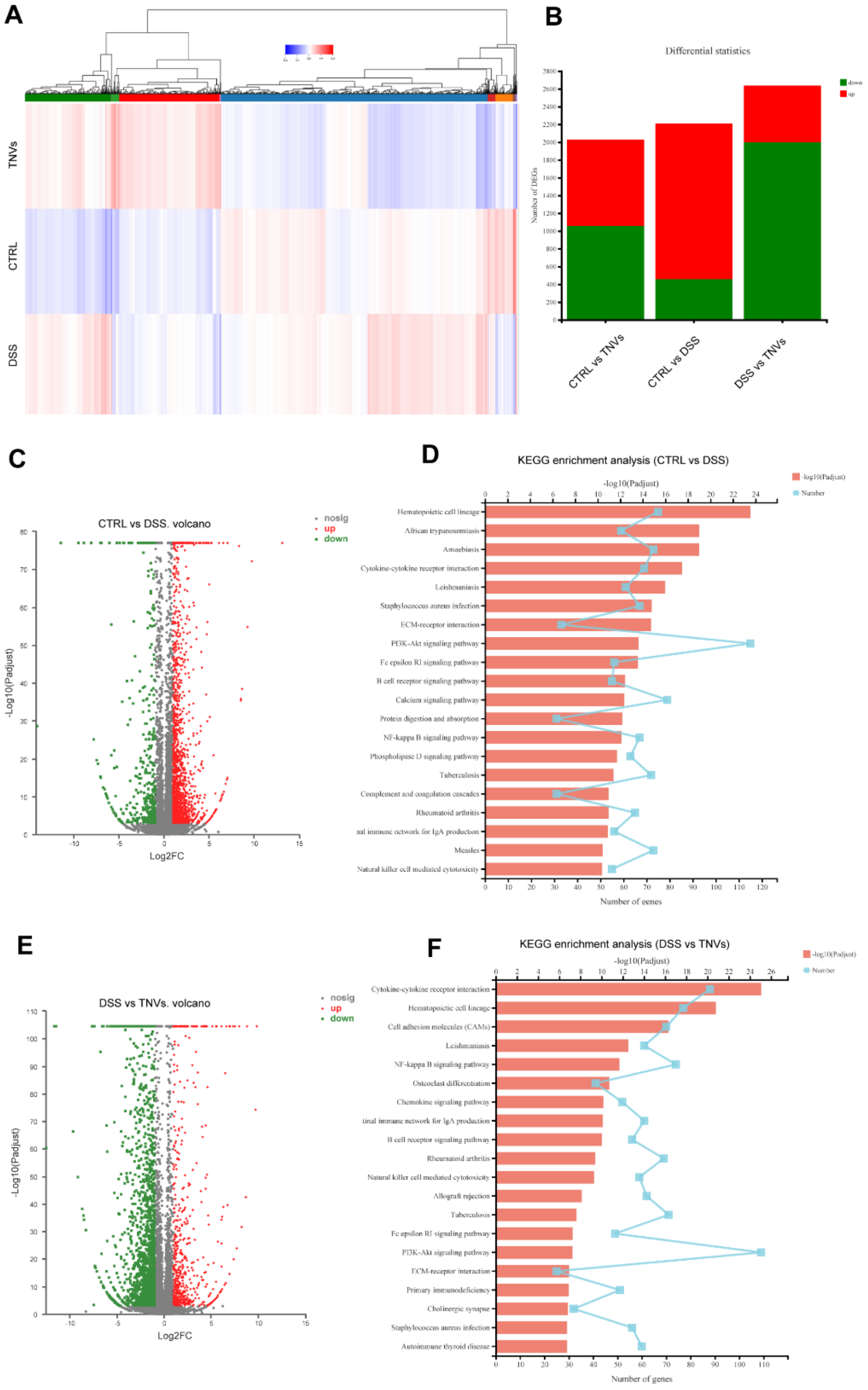

Figure S8. The concentration of serum biochemical factors. Alanine aminotransferase (ALT), aspartate aminotransferase (AST), blood urea nitrogen (BUN), creatinine (CREA) in the serum of chronic colitis mice experienced from different treatment, n = 5.

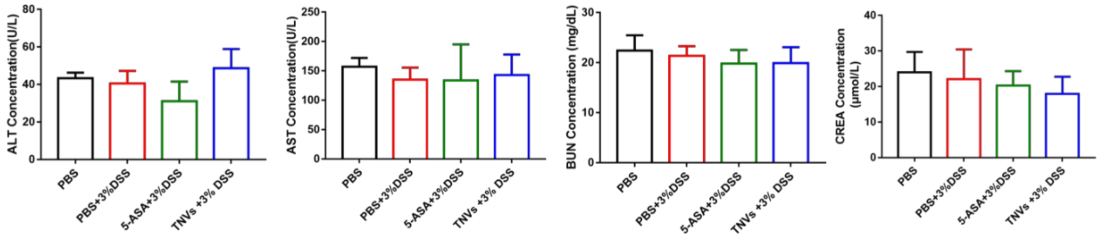

Table S1. The size diameter, Polydispersity index and Zeta potential of TNVs from three different bands.

Samples	Size diameter (nm)	Polydispersity index	Zeta potential (mV)
TNVs 8-30%	191.7 ± 15.83	0.22 ± 0.02	-13.53 ± 0.93
TNVs 30-45%	243.9 ± 13.99	0.19 ± 0.02	-17.5 ± 1.15
TNVs 45-60%	800.5 ± 66.63	0.61 ± 0.21	-9.65 ± 0.36

Table S2. The yield of TNVs in the different bands, protein concentration (mg) per fresh turmeric (kg), n = 3.

Samples	Yield (mg/kg)
TNVs 8-30%	98.61 ± 12.88
TNVs 30-45%	55.84 ± 7.64
TNVs 45-60%	9.73 ± 2.33

Table S3. The content of curcuminoids in three different bands using LC-MS, n = 3, mg/kg fresh turmeric.

Samples	Bisdemethoxycurcumin	Demethoxycurcumin	Curcumin
TNVs 8-30%	5.28 ± 0.78	5.00 ± 0.84	15.96 ± 3.26
TNVs 30-45%	2.78 ± 0.92	2.63 ± 1.09	8.36 ± 2.17
TNVs 45-60%	0.54 ± 0.19	0.55 ± 0.19	3.30 ± 0.23
